# Environmental impact of dental amalgam and alternative restorative materials: a systematic review

**DOI:** 10.1038/s41405-026-00399-z

**Published:** 2026-01-21

**Authors:** Simon Briscoe, Liz Shaw, Hassanat Mojirola Lawal, Clara Martin Pintado, Noreen Orr, Lauren Asare, G. J. Melendez-Torres, Ruth Garside, Jo Thompson Coon

**Affiliations:** 1 Exeter Policy Research Programme Evidence Review Facility, University of Exeter Medical School, University of Exeter, Exeter, Devon UK; 2 European Centre for Environment and Human Health, Department of Public health and Sports Science, University of Exeter, Penryn, Cornwall UK

**Keywords:** Restorative dentistry, Dental materials

## Abstract

**Introduction:**

Concern about the environmental impact of dental restorative materials has grown in recent years. The most common material for dental restorations has historically been amalgam, but this has seen a decline due to concerns about the health and environmental impact of mercury. Alternative dental restorative materials include resin-based composite (RBC) and glass-ionomer cement (GIC), but both have uncertain environmental impacts. This systematic review aimed to compare the environmental impact of dental amalgam versus other restorative materials used for direct restorations.

**Methods:**

We undertook a systematic review which followed established guidance and prospectively registered our protocol on PROSPERO (CRD42024608563). Searches of bibliographic databases included Environment Complete, GreenFILE, Science Citation Index, CAB Abstracts, MEDLINE and CINAHL. These were supplemented with checking reference lists and forward citation searching of included studies. Retrieved records were screened by two independent reviewers at title and abstract and full text using pre-defined inclusion criteria. Data extraction and quality appraisal were undertaken by one reviewer and checked by a second. Findings were narratively synthesised.

**Results:**

Twenty-one studies (*n* = 22 study reports) were included. Eleven were in a clinic setting, three were in a lab setting, five were in both clinic and lab settings and two were in a crematorium setting. Six studies included a comparison between different restorative materials, the remainder only measured environmental impact of one material (or multiple without comparison). Some studies were framed as potential environmental impact due to limited data. Materials included dental amalgam, RBC and GIC. Studies were highly heterogenous which limited the scope for synthesis of findings. All materials were associated with environmental impact.

**Conclusion:**

Whilst the evidence included in this review indicates that both amalgam and non-amalgam dental materials are associated with environmental impacts, confidence in these findings is limited due to the heterogeneity of study designs, outcome measures, and limited comparative data. Further research is needed to inform future policies which aim to reduce the environmental impact of restorative dental activities.

## Introduction

In the last 10 years, concern about the health and environmental impact of dental restorative materials has grown. The most common material for dental restorations has historically been amalgam, but this has seen a decline in recent years due to concerns about the health [1] and environmental impact of mercury [2]. Alternative dental restorative materials include resin-based composite (RBC) and glass-ionomer cement (GIC), which have seen a corresponding increase in use, but there are also concerns about the health and environmental impact of these materials—including constituent and trace substances such as Bisphenol A (BPA) and monomers, which can cause harms to humans and the wider natural world [3, 4]. The level of toxic substances in dental restoration materials may be small, but the sheer scale of restorations could potentially have significant health and environmental impacts. A 2018 survey of adults in England (*n* = 14,270) showed that 90.2% had restorations in an average of 7.2 teeth [5]. In particular, direct dental restorations are a common type of restoration, and make use of the main types of material which are causing concern for health and environmental impacts, including dental amalgam, RBC and GIC [6, 7]. Concerns relate to leaching of toxic chemicals across the life course of restorative material, and also during the processes of placement, removal and disposal [8].

Despite the uncertainty about alternatives to dental amalgam, in the last ten years the trajectory in dental restorations has been to move away from amalgam to other types or restorative material. This has been accelerated by the 2013 *Minamata Convention on Mercury*, which is a United Nations (UN) global treaty adopted by 147 countries including the United Kingdom (UK) that aims to protect human health and the environment from the adverse affects of mercury [9]. In this context, the UK is considering phasing out the use of dental amalgam. However, there is a need to assess the evidence of harm across different types of material to make an informed decision. In particular, the environmental impact of these materials is relatively under-studied compared to the health impact. Whereas there are at least three systematic reviews which have considered the health impacts of dental amalgam versus alternative restorative materials [10–12], to date there is only one review, now seven years old, which attempts compare the environmental impacts of dental amalgam and alternative restoration materials [11]. This review by the Canadian Agency for Drugs and Technologies in Health (CADTH) concluded that a detailed comparison of the environmental effects of dental amalgam and RBC materials was not possible due to a lack of relevant studies [11]. Overall, the review had broad inclusion criteria which included literature reviews [13], commentaries [14, 15], and reports which use a mix of case studies, literature reviews and expert opinion [16, 17].

We were commissioned by the UK National Institute for Health and Social Care Research (NIHR) Policy Research Programme to carry out a systematic review of studies which compare the adverse environmental impacts of dental amalgam versus other dental restorative materials, with a view to informing decisions on the future use of dental amalgam within the UK. The commissioning brief for the review included a request to consider both health and environmental impacts. The health-related impacts are presented in a separate report (in press). In this paper, we present the methods and findings of this review as they relate to the environmental impacts of dental amalgam versus alternative restorative materials.

## Methods

We prospectively registered our systematic review protocol on PROSPERO (https://www.crd.york.ac.uk/PROSPERO/view/CRD42024608563) [18]. The methods used to conduct and report the findings of the review followed recommended conduct [19] and PRISMA reporting guidance [20].

### Search strategy

We searched a selection of bibliographic databases including MEDLINE and CAB Abstracts (both via Ovid), Environment Complete, CINAHL and GreenFILE (all via ProQuest), and the Science Citation Index (via Web of Science). All search strategies are reproduced in supplementary material. We used a 2007 date-limit with the same rationale as the most recent systematic review to ensure findings are relevant to the contemporary context [11]. We also applied an English language limit. In addition, we undertook backward and forward citation searches of studies which met the inclusion criteria and checked the included studies of relevant systematic reviews. Furthermore, we inspected the Healthcare LCA database (https://healthcarelca.com/).

### Study identification

Two reviewers independently screened all records retrieved using EPPI-Reviewer 6 (EPPI Centre, UCL Social Research Institute, University College London). The following inclusion criteria were applied: Population/setting: Settings in which the environmental impact of dental materials are measured, e.g., clinics, crematoriums, natural world including animal and plant studies, lab-based settings; Intervention: Direct restorations or extractions/incineration of direct restorations, including with respect to the following materials: dental amalgam, RBC, GIC, and resin modified glass ionomer cements. (Studies which included non-relevant materials/interventions were included if they also included relevant materials/interventions); Comparator: Any comparator or none; Outcome: All environmental outcomes including (but not limited to): levels of toxicity including mercury, BPA, monomer concentration; global warming potential, carbon-emission data; water toxicity, ecotoxicity, human and animal toxicity; Study design: Prospective/retrospective cohort studies; cross-sectional studies; pre-post studies; life cycle assessments; randomised controlled trials; controlled trials. Studies were also limited to high income countries according to the World Bank List to facilitate comparison with similar dental service settings [21]. Studies were excluded if they were health impact studies, only included dental materials not used in direct restorations (e.g., ceramics, metal alloys, porcelain, CAD/CAM materials), did not report data relating to direct restorations or extractions/incineration of direct restorations, were case studies or systematic reviews/literature reviews, or reported data from non-high income countries. Disagreements were resolved through discussion or referral to a third reviewer. Full-texts were screened in the same way.

### Data extraction and quality appraisal

One reviewer extracted data from each study which was then checked by a second reviewer. We undertook quality appraisal using the Collaboration for Environmental Evidence Critical Appraisal Tool (CEECAT) Version 0.3 [22]. CEECAT is a critical appraisal tool which has been developed for use in systematic reviews with an environmental focus. The tool is still under development but is in its third iteration (version 0.3) and available for use. The tool assesses risk of bias in studies across several domains, including confounding, selection of subjects for study, deviations from intended intervention or exposure, missing data, measurement of outcomes and reporting of findings [23]. Based on the risk of bias across these domains the tool provides an overall risk of bias assessment, which determines whether a study is high, medium or low risk of bias [23]. Quality appraisal was undertaken by one reviewer and checked by a second reviewer.

### Synthesis of the evidence

We grouped studies according to the type of restoration material evaluated, including groups for studies which included multiple restorative materials, and groups for studies which only included one type of restorative material. Within these groups, we categorised studies within impact categories which described the type of environmental impact that was assessed. These categories were inductively derived from the included studies with reference to the outcomes which were measured. The narrative synthesis sought to identify and explain, where possible, patterns in the data regarding the environmental impact of the restorative materials. Units were standardised where possible. Impact was assessed in terms of the degree to which exposure to dental restorative materials was associated with environmental harms. This included both harm to the natural environment, and human exposure to harmful levels of toxic substances in the immediate environment of activity relating to restorative materials which was not otherwise related to health conditions.

Due to limitations in how environmental studies reported and analysed their data, it was not always clear whether the outcomes they described translated into actual environmental impact. Specifically, this occurred where a study only reported levels of toxic discharge (e.g., in wastewater/emissions/particulate matter) during dental restorative procedures, without assessing how these emissions impact the environment. We included studies reporting this more limited data because, although the impact was not evaluated, the findings remained broadly relevant to understanding environmental effects. This decision was also influenced by the relatively small number of studies identified which measured environmental impact. Studies which only reported levels of toxicity with no further analysis were reported as indicative of *potential* environmental impact.

To show *actual* environmental impact, studies were required to either:

- report compliance (or not) with guidance on levels of toxicity in the environment associated with restorative materials, or
- report a measure of environmental impact, such as the effect of toxicity on the environment (e.g., animal toxicity) or carbon footprint.

With respect to (a) compliance with guidance, we inspected the results and discussion sections of included studies to identify whether findings were reported as compliant with recommended toxicity thresholds. Whether or not studies comply with guidance, or report a measure of environmental impact such as animal toxicity, is indicated in the narrative synthesis. In addition, the final section of the narrative synthesis presents a tabulated summary of studies that assessed environmental impact through one or more of these approaches.

### Interest holder involvement

We consulted with and worked closely alongside interest holder groups throughout the review. Interest holders included those requesting the review from the Department of Health and Social Care (DHSC), people with expertise in dental restoration and the Cochrane Oral Health team. The review also benefited from interactions with PERSPEX, a group of 14 public collaborators who bring their carer, patient and public perspective to the work of the Exeter PRP Evidence Review Facility. Membership is culturally, geographically and demographically diverse (https://www.exeter.ac.uk/research/groups/medicine/esmi/workstreams/perspex/).

### Protocol deviations

Due to the low number of studies meeting our inclusion criteria, we included lab-based studies, including in vitro studies.

## Results

### Search and study identification

**Fig. 1:**
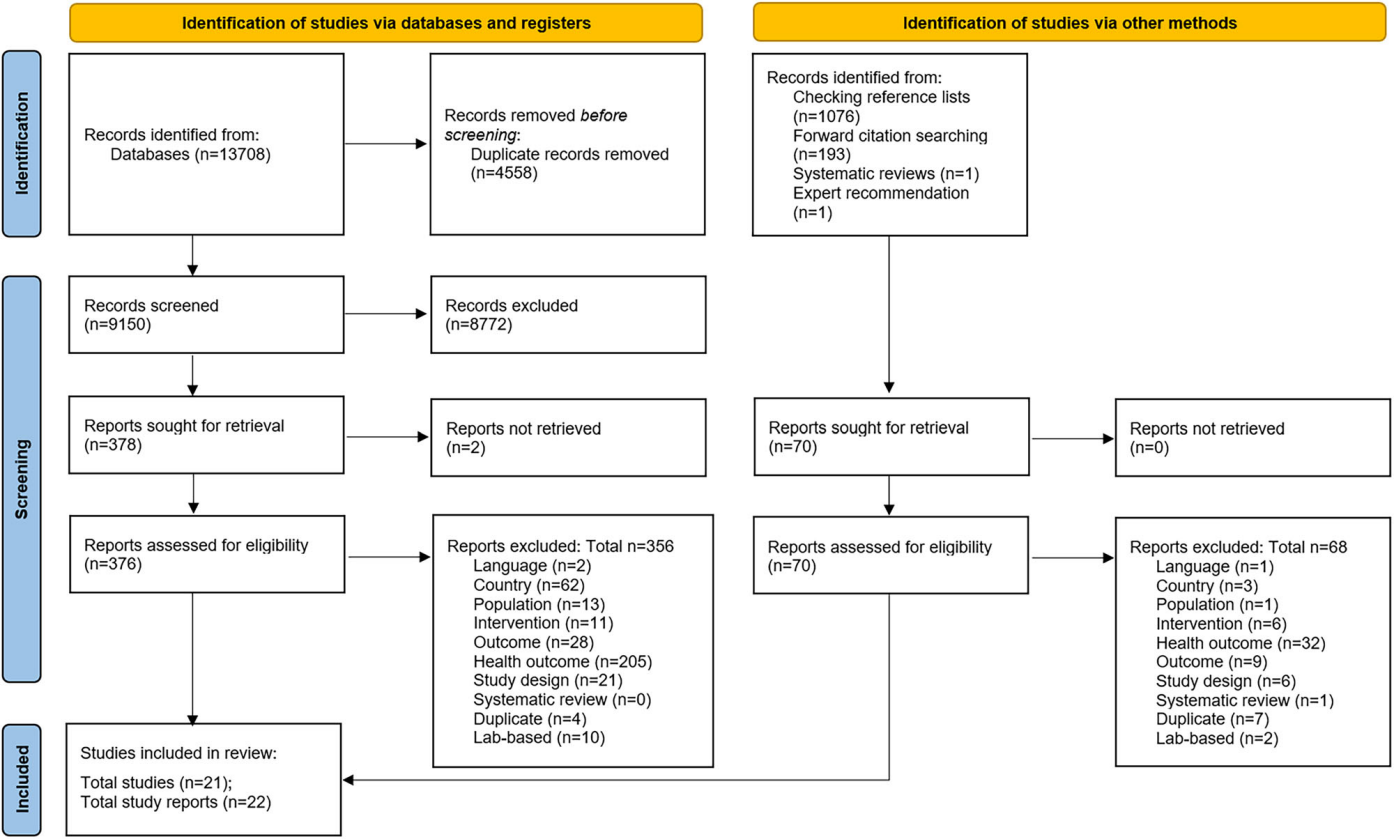
PRISMA flow diagram. Diagram showing the identification, screening, reasons for exclusion at full-text and number of included studies for the systematic review.

Bibliographic database searches identified 13,708 records in total on 6^th^ November 2024. There were 9150 following de-duplication. Of these, 378 full-texts were sought for full-text screening of which 376 were successfully retrieved. This includes the number which met the inclusion criteria for both the health and environmental components of the overarching research request, of which only the latter are the focus of the present paper. An additional full-text study was recommended during peer review [24]. In total, 21 studies published across 22 study reports were included in the review [24–45]. Of these, 21 were published in journal article format and one was published as a grey literature report. The one grey literature report [24] is a sibling study report to one of the journal articles [26]. This process is depicted in the PRISMA flow diagram in Fig. 1. Additionally, one study report contained a two-part study which is herein counted as one study, but each part was separately quality appraised and appears in separate sections in the presentation of results [31].

### Key characteristics of included studies

Key characteristics of individual studies are summarised in Table 1. Overall characteristics are summarised in Table 2. Inspection of the key characteristics and outcomes reported in the included studies determined that they were too heterogenous for meta-analysis, hence we used narrative synthesis.

**Table 1:** Key characteristics of included studies.

Study [Country]	Study design	Setting	Restorative material [Activity]	Category
Al-Kawas 2008 [United Arab Emirates]: JAP [25]	Cross-sectional with comparator	Clinic	Amalgam, composite, glass-ionomer [Restorations, removals]	Mercury levels in wastewater
Binner 2022 [Ireland]; JAP [26]; Harding 2022 [Ireland] GLR [24]	Cross-sectional	Clinic	Composite, glass-ionomer, resin-modified glass ionomer cements [NR]	Animal toxicity of restorative materials
Duane 2017 [UK]; JAP [27]	Secondary analysis of dataset	Clinic	Amalgam, composite, glass-ionomer [Restorations]	Carbon footprint of restorative materials
Gioda 2007 [USA]; JAP [28]	Before and after study	Clinic and lab	Amalgam [Restorations, removals]	Mercury levels in vapour
Kim 2018 [South Korea]; JAP [29]	Cross-sectional	Clinic	Amalgam [Incineration]	Mercury levels in emissions
Kontogianni 2008 [Greece]; JAP [30]	Cross-sectional survey	Clinic	Amalgam [NR]	Mercury levels in solid waste
Majstorovic 2024 [Croatia]; JAP [31]	Cross-sectional (clinic setting) and controlled trial (lab-based)	Part 1: clinic; part 2: lab	Part 1: NR [NR]; Part 2: Commercial composite, glass-ionomer, laboratory-prepared composite, alkasite, amalgam [Leaching]	Animal toxicity of restorative materials
Mandalidis 2008 [Greece]; JAP [32]	Cross-sectional	Clinic	Amalgam [Restorations, removals]	Mercury levels in solid waste
Marquardt 2009 [Germany]; JAP [33]	Cross-sectional	Clinic	Composite [Restorations]	Monomer levels in emissions
Mourouzis 2022 [Greece]; JAP [34]	Controlled trial	Clinic and lab	Composite, polymer infiltrated ceramic CAD/CAM [Restorations]	Monomers levels in wastewater
Olivera 2020 [USA]; JAP [35]	Prospective longitudinal	Clinic	Amalgam [Restorations]	Mercury levels in wastewater and in solid waste
Paryag 2010 [Trinidad and Tobago]; JAP [36]	Modelling study	Lab	Amalgam [Removals]	Mercury levels in wastewater
Piagno 2020 [Canada]; JAP [37]	Modelling study	Crematorium	Amalgam [Incineration]	Mercury levels in emissions
Polydorou 2023 [Germany]; JAP [38]	Controlled trial	Lab	Composite [Dental grinding procedures]	Monomer levels in wastewater
Shraim 2011 [Saudi Arabia]; JAP [39]	Prospective longitudinal with comparator	Clinic	Location 1&2: Amalgam or composite [Restorations], glass ionomer [endodontic treatment]; Location 3a + 3b:Amalgam, composite or glass-ionomer [Restorations]; Location 3c: Composite [Restorations]	Mercury levels in wastewater
Simu 2014 [Romania]; JAP [40]	Cross-sectional	Clinic	Amalgam, composite, glass-ionomer [ultrasound scaling, root canal preparation, cavity preparation and filling, removal of silver amalgam, composite, glass-ionomer filings, teeth preparation for prosthetic purpose, orthodontic controls, etc.]	Mercury levels in particulate matter
Stone 2009 [USA]; JAP [41]	Prospective longitudinal with control	Clinic and lab	Dental amalgam [NR]	Mercury levels in wastewater
Takaoka 2010 [Japan]; JAP [42]	Modelling study	Crematorium	Amalgam [Incineration]	Mercury levels in emissions
Van Landuyt 2014 [Belgium]; JAP [43]	Controlled trial	Clinic and lab	Composite [grinding material to simulate abrasive procedures, including restorations]	Monomer levels in particulate matter
Warwick 2013 [Canada]; JAP [45]	Controlled trial (lab based)	Lab	Amalgam [Removals]	Mercury levels in vapour
Warwick 2019 [Canada]; JAP [44]	Prospective longitudinal with control	Clinic	Amalgam [Removals]	Mercury levels in vapour

*GLR* grey literature report, *JAP* journal article publication, *NR* not reported (several studies do not report the type of dental activity which was carried out, and one study does not report the type of material which was used), *USA* United States of America.

**Table 2:** Overall characteristics of included studies.

Characteristics	*n*
*Study design*	
Before and after	1
Controlled trial	5
Cross-sectional	8^a^
Modelling/simulation	3
Prospective longitudinal	4
Secondary analysis	1
*Settings*	
Clinic only	11
Lab only	3
Clinic and lab	5
Crematorium	2
*Restorative material*^b^	
Dental amalgam	17
Resin-based composite	10
Glass-ionomer cement	6
Alkasite	1
*Treatment activity*	
Restorations	10
Removals	8
Incineration of extracted teeth	3
Not reported	4

^a^Includes one study which also had a controlled trial component [31].

^b^Some studies included more than one type of restorative material.

### Quality appraisal

Quality ratings for each individual study are displayed in supplementary material (see Table S1 in supplementary material). Overall, eleven of the 21 included studies (including Majstorovic et al. part 1) [25–29, 31, 32, 36, 37, 39, 41] had a ‘high’ risk of bias and 11 studies (including Majstorovic et al. part 2) had a ‘medium’ risk of bias [30, 31, 33–35, 38, 40, 42–45]. Majstorovic et al. included a two part study, each of which was quality appraised separately [31]. No studies were classified as ‘low’ risk of bias.

### Narrative synthesis

Studies which included a comparison between different types of restorative material are presented first, followed by non-comparative studies, i.e., studies which only included one type of restorative material (either dental amalgam or RBC) or multiple types with no comparison between materials. Within each section for comparative and non-comparative studies, the studies are grouped within impact categories (see Table 3). There are 12 impact categories, including one (animal toxicity) which appears in more than one grouping of restorative materials. Supplementary tables provide the data underpinning the narrative synthesis, while the narrative synthesis itself includes all information needed for interpreting the evidence. Tables prefixed with an S (e.g., Table S2) are in supplementary material.

**Table 3:** Restorative material groupings and impact categories.

Type of material, *n* = number of studies	Impact categories, *n *=number of studies
Studies which included multiple restorative materials with comparison (*n* = 6):	Toxicity levels in wastewater (*n* = 2) [24, 25, 39]
	Toxicity levels in particulate matter (PM) (*n* = 1) [40]
	Animal toxicity (*n* = 1) [31]
	Carbon footprint (*n* = 1) [27]
Studies which included multiple restorative materials with no comparison (*n* = 2)	Animal toxicity (*n* = 2) [26, 31]
Studies which only included dental amalgam restorative material (*n* = 11):	Mercury levels in wastewater (*n* = 3) [35, 36, 41]
	Mercury levels in solid waste (*n* = 3) [30, 32, 35]
	Mercury levels in emissions (*n* = 3) [29, 37, 42]
	Mercury levels in vapour (*n* = 3) [28, 44, 45]
	Mercury levels in PM (*n* = 1) [28]
Studies which only included resin-based composite restorative materials (*n* = 4):	Monomer levels in wastewater (*n* = 2) [34, 38]
	Monomer levels in PM (*n* = 1) [43]
	Monomer levels in emissions (*n* = 1) [33]

Finally, in this section, we show in tabulated format the findings from studies where environmental impact was measured (i.e., actual environmental impact) versus studies where there was only potential environmental impact.

### Multiple restorative materials with comparison (*n* = 6)

Six studies (*n* = 7 study reports) included a comparison between different restoration materials [24–27, 31, 39, 40]. These studies were categorised within four impact categories: toxicity levels in wastewater (*n* = 3 [*n* = 4 study reports]) [24–26, 39]; toxicity levels in particulate matter (*n* = 1) [40]; animal toxicity (*n* = 1) [31]; and carbon footprint (*n* = 1) [27]. All studies included dental amalgam in the comparison with other materials.

#### Toxicity levels in wastewater in studies which compare restorative materials (n = 3)

Al Kawas et al. [25] and Shraim et al. [39] compared mercury levels in wastewater for different dental restorative materials, both including amalgam (see Table S2). Both were in clinic settings and classified as high risk of bias. Mercury levels in Al Kawas et al. were highest in samples from clinics where only amalgam restorations were performed, but was still present in samples when no amalgam was used due to sedimentation of amalgam particles from previous treatments inside wastewater tubes [25]. No statistical testing was undertaken to assess the significance of the results, nor assessment of compliance with wastewater guidance.

Similarly, mercury levels in Shraim et al. were highest in the clinic where the most dental amalgam restorations were carried out, but still present where no amalgam restorations were performed [39]. The authors suggest the latter finding is likely due to dental amalgam removals taking place in this clinic, which are replaced by RBC fillings [39]. The study reports that hazardous levels of amalgam were found in most samples according to the Technical Regulations for Discharge of Raw Wastewater of the Kingdom of Saudi Arabia (KSA); also hazardous levels of Mg, Mn, Cu, Zn, Sn and Ba, some of which are amalgam constituents [39]. Negligible levels of metal concentration were found in the inlet water. Statistical significance of findings was not assessed. Notably, the clinics sampled in both Al Kawas et al. [25] and Shraim et al. [39] were reported to not use amalgam separators.

One study (*n* = 2 study reports) reported total suspended solids and total dissolved solids derived from dental amalgam, RBC and GIC restoration activity respectively across three clinics (see Table S2) [24, 26]. In one clinic, the total suspended solids were reported separately for each material and compared. This showed that levels of total suspended solids derived from RBC and GIC materials were higher than permissible limits according to the Protection of the Environment Act (2003) and EU Directive 91/271/EEC [24]. The highest levels related to GIC material. Contrastingly, levels of total suspended solids were within limits for amalgam but above amalgam limits for total dissolved solids [24]. Unlike Al Kawas et al. [25] and Shraim et al. [39], the clinics in this study did use amalgam separators, and the study highlights how toxic discharge from mercury free restoration materials are not filtered effectively by separators [24]. It was not clear, however, exactly how much of each restoration material was used in the clinics [24, 26]. The study was rated as high risk of bias.

#### Toxicity levels in particulate matter in studies which compare restorative materials (*n* = 1)

One study compared mercury levels in particulate matter for different dental restorative materials in a clinic setting, including amalgam (see Table S3) [40]. Simu et al. reported that silver amalgam restorations contained more toxic compounds than other types of restoration material, including RBC and GIC [40]. However, the study did not report the relative degree to which amalgam and non-amalgam restorative materials contain toxic particles and whether these were at harmful levels [40]. Compliance with guidelines was also not reported. The study was rated medium risk of bias.

#### Animal toxicity in studies which compare restorative materials (*n* = 1)

One study compared the animal toxicity of mercury and monomer levels in wastewater associated with dental amalgam and RBC respectively (see Table S4) [31]. This study was conducted in a lab setting and rated medium risk of bias. Majstorovic et al. assessed toxicity of mercury and monomers released into water through leaching of dental amalgam and RBC materials for zebrafish [31]. The highest toxicity to zebrafish was caused by experimental composite, followed by dental amalgam, commercial RBC, GIC and alkasite. Toxicity reduced after 7 days. Developmental abnormalities were statistically significant for dental amalgam and experimental composite, including at LC_30_ values for both incubation periods. Developmental abnormalities were not statistically significant for other restorative materials. It was unclear whether amalgam separators were used or simulated in this study, which may mean that mercury levels in the comparison were higher than they would be in a real-world setting where amalgam separators were used.

#### Carbon footprint in studies which compare different restorative materials (*n* = 1)

Duane et al. was the only study to compare the carbon footprint of different restoration materials (see Table S5) [27]. This related to clinic settings, i.e., carbon footprint associated with dental activities in NHS primary care dental clinics in England. Duane et al. reported the total carbon footprint for dental amalgam and RBC restorative materials was similar, with amalgam slightly higher [27]. Total carbon footprint for GIC was lowest of the three materials, but also accounted for the smallest proportion of dental services (1.5%) compared to dental amalgam (9.79%) or RBC (9.52%) restorative treatment [27]. Per procedure, glass-ionomer restorations had just over half the carbon footprint of dental amalgam or RBC (8.58 vs 14.76 vs 14.75 kgCO2e respectively) [27]. However, Duane et al. also noted that most of the data for calculating carbon footprint of procedures was based on time and energy use, and that there were no data available relating to the materials themselves [27]. The study was rated high risk of bias.

### Multiple restorative materials with no comparison (*n* = 2)

Two studies (*n* = 3 study reports) included multiple restorative materials with no comparison between materials (see Table S6) [24, 26, 31]. Both studies assessed animal toxicity relating to wastewater in clinic settings. One study included only non-amalgam materials in the analysis [24, 26] and one study did not report the materials which were included [31].

#### Animal toxicity in studies which include multiple materials (with no comparison) (*n* = 2)

Majstorovic et al. found that mercury toxicity was statistically significant for zebrafish exposed to samples from one of three clinics with the highest mercury concentration (*p* < 0.001) [31]. Although no zebrafish mortality occurred, developmental abnormalities were observed, and no hatching was recorded in embryos exposed to samples from the clinic with the highest mercury concentrations. No monomers were detected in the wastewater. In Binner et al., 48-h EC₅₀ values for *Daphnia magna* exposed to wastewater samples from non-amalgam restorative materials—including RBC and GIC—ranged from 0.2 to 32.9 mL/L [26]. The variation was attributed to differing levels of disinfectants in the wastewater [26]. A sibling study report showed that levels of total suspended solids relating to RBC and GIC materials in the wastewater were higher than permissible limits according to the Protection of the Environment Act (2003) and EU Directive 91/271/EEC (see above: *Toxicity Levels in Wastewater*) [24]. Binner et al. interpreted their finding as showing environmental impact associated with non-amalgam materials despite the preference for using these instead of dental amalgam due to the absence of mercury [26]. Both studies were rated as high risk of bias.

### Dental amalgam only (*n* = 11)

Eleven studies included dental amalgam restoration material only [28–30, 32, 35–37, 41, 42, 44, 45]. These studies were categorised within five impact categories: mercury levels in dental wastewater (*n* = 3) [35, 36, 41]; mercury levels in solid waste (*n* = 3) [30, 32, 35]; mercury levels in emissions (*n* = 3) [29, 37, 42]; mercury levels in vapour (*n* = 3) [28, 44, 45]; and mercury levels in PM (*n* = 1) [28].

#### Mercury levels in wastewater in studies which include dental amalgam only (*n* = 3)

Three studies considered mercury levels in wastewater (see Table S7) [35, 36, 41]. These were a heterogeneous set of studies with limited scope for comparison. Olivera et al. assessed mercury levels in wastewater from chairside amalgam separators (CAS) (*n* = 6) as part of ISO 11143:200820 testing [35]. The CAS units were found to perform better than the US Environmental Protection Agency (EPA) standard with respect to mercury levels in wastewater—albeit, this related to overall performance of the CAS rather than specifically focusing on mercury levels [35]. No statistical analysis of the significance of findings was undertaken. The study was rated medium risk of bias.

Paryag et al. used water collected from a lab-based simulation study of dental amalgam restorations to calculate an estimate of the daily and monthly amount of mercury discharge in wastewater generated by dentists in Trinidad and Tobago [36]. They included both liquid and solid portions of mercury filtrate in the lab-based simulation to estimate the total amount of mercury discharge. No statistical analysis or comparison with guidance was reported. However, they compared the estimate with previous estimations of daily mercury waste from Canadian dentists, which was found to be much lower despite no amalgam separators used in Trinidad and Tobago [36]. The study was rated high risk of bias.

Stone et al. compared the degree to which chlorine and chloramine mobilise mercury from dental amalgam, including both lab prepared amalgam and samples obtained from dental wastewater [41]. This was done to assess the relative mercury levels following switch to chloramine in many water treatment facilitates in the USA. The study found that chloramine mobilises mercury to a lesser degree than chlorine (*p* < 0.001). The study was rated high risk of bias.

#### Mercury levels in solid filtrate/solid waste in studies which include dental amalgam only (*n* = 3)

Kontogianni et al. [30] and Mandalidis et al. [32] reported amalgam waste generated by dental clinics in two different regions of Greece, with a seven-year gap between data collection periods (2006–2013 respectively) (see Table S8). Kontogianni et al. was rated medium risk of bias [30] and Mandalidis et al. rated high risk of bias [32]. The proportion of total waste comprising amalgam was lower in the more recent study, potentially reflecting the declining use of dental amalgam over time [32]. Although both studies linked amalgam waste with mercury, neither assessed mercury concentrations in the waste nor evaluated waste levels against waste management guidelines.

In a different context, Olivera et al. reported the mean total solids accumulated in each CAS (*n *= 6) at the end of service life [35]. Overall, the CAS units assessed in the study were found to perform better than the USA EPA standard; albeit, this related to overall performance of the CAS rather than specifically focusing on mercury levels [35]. The study was rated medium risk of bias.

#### Mercury levels in emissions in studies which include dental amalgam only (*n* = 3)

Two studies measured mercury concentrations in crematorium emissions, one was rated high risk of bias [37] and the other medium risk [42]. Both studies used modelling approaches to estimate the average annual mercury emissions from the incineration of extracted teeth during cremation—one in British Columbia, Canada [37], and the other in Japan [42]. Although the estimated total annual mercury emissions were similar (approx. 35 kg/year), and both studies assumed approximately 50% mercury content per filling, the estimated mercury released per cremation varied dramatically: Takaoka et al. estimated 31.2 mg per cremation [42], while Piagno et al. estimated 2.1 g per cremation, around 6400% higher value [37]. This discrepancy likely reflects differences in model assumptions, such as amalgam prevalence, pre-cremation tooth removal, and population dental history. The difference in the overall percentage of national mercury emissions attributed to crematoriums ( > 7% in BC vs <0.01% in Japan) may be due to industrial and demographic factors, including Japan’s much larger industrial economy [46].

In a different context, Kim et al. reported total mercury emissions from incineration of extracted teeth at Korean dentistry units (see Table S9) [29]. The study was rated high risk of bias. They did not report whether this met recommended guidance, but they state that Korean guidelines did not (at time of writing) require separating amalgam from non-amalgam restorations before incinerating as required in the USA, and they recommend that this practice is introduced [29]. They also cited a trend for reduction in dental amalgam restorations in Korea in the last decade (4.1 million in 2012 to 3.54 million in 2013 and 3.09 million in 2014) [29].

#### Mercury levels in vapour in studies which include dental amalgam only (*n* = 3)

The two studies in clinic settings in this category (including one which had a clinic component alongside a separate lab component) both assessed findings with reference to the USA Occupational Safety and Health Administration (OSHA) permissible exposure limit (100 μg/m^3^) (see Table S10) [28, 44]. Gioda et al. [28] was rated high risk of bias and Warwick et al. [44] medium risk of bias [44]. Gioda et al. reported mercury concentrations in vapour released from dental amalgam restorations and removals during a third-year dental student examination, using personal vapour samplers worn by students (*n* = 23) [28]. The mean mercury exposure level was 50.6 μg/m³ ( ± 9.7 SE), which is below the OSHA permissible exposure limit but slightly above the advisory time-weighted average (TWA) recommended by the American Conference of Governmental Industrial Hygienists (ACGIH) for an 8-h day/40-hour week (50 μg/m³). However, students were only exposed for approximately 4.2 h on a single day [28]. No mercury vapour was detected in the room prior to the examination.

Warwick et al. [44] reported mercury vapour concentrations during dental amalgam extractions for 21 patients across two clinics, alongside two control patients [44]. Mercury levels were recorded as peak values as well as 15-, 30-, and 60-min averages for each procedure (see Table S10). In contrast to Gioda et al. [28], Warwick et al. observed mean peak concentrations exceeding the OSHA permissible exposure limit (195 μg/m³, SD = 241) [44]. However, this refers to peak levels, whereas Gioda et al. reported a mean exposure over a 4.2-h period. Although levels declined over the subsequent hour, the mean 60-min concentration remained above the OSHA limit at 109 μg/m³. Warwick et al. further noted that the 60-min average was high enough that, even assuming no further exposure for the remaining 7 h of an 8-h workday, the time-weighted average would still exceed the ACGIH’s recommended limit of 25 μg/m³ [44].

Gioda et al. reported mercury concentration in vapour from dental amalgam restorations and removals during a dental student exam in a lab setting using vapour samplers attached to students (*n* = 5) [28]. They reported that some students were exposed to mercury concentrations 30 times above the OSHA’s recommended level of 100 μg/m^3^ [28]. They noted this was higher than in the clinic setting exam because of the longer sampling period and higher number of amalgam capsules used. Also, the students in the clinic setting were third year students versus second-year students in the lab setting who were less experienced [28].

Warwick et al. [44] reported mercury vapour concentrations during a laboratory simulation of removal of dental amalgam restorations from teeth under three different conditions [44]. When both suction and water spray were used, mercury concentrations remained below the Alberta Occupational Ceiling Limit of 125 μg/m³. In contrast, this limit was exceeded during 8% of the suction-only test duration and 16% of the time in the no-suction/no-spray condition [44]. The Alberta Occupational Threshold Limit Value (AOTLV) of 25 μg/m³ (8-hour time-weighted average) was breached in 100% of readings under the no suction or water condition, 84% of readings with suction only, and 0% with water spray and suction [44]. The study was rated medium risk of bias.

#### Mercury levels in particulate matter (*n* = 1)

Gioda et al. reported mercury concentration in PM from dental amalgam restorations and removals during a one-day dental student exam (*n* = 35 students) [28]. The average concentration of Hg bound to PM_10_ was 0.1 μg/m3 (0.01–0.2 μg/m3) (see Table S11). They reported that there were no recommended PM_10_ indoor air quality standards, but total dust should not exceed 100 μg/m^3^ [28]. Gioda et al. undertook the same test in a lab environment, for a different set of students (*n* = 39) [28]. The average concentration of mercury bound to PM_10_ was nine times higher at 0.6 μg/m3 (0.1–1.2 μg/m^3^). The highest mercury concentrations occurred during the day of the exam. They reported that the higher concentrations of mercury were associated with students needing to repeat restorations which were not properly performed, and may be higher than in the dental clinic due to the students being less experienced than the clinic students [28]. This study was rated high risk of bias [28].

### Resin-based composite only (*n* = 4)

Four studies included RBC material only [33, 34, 38, 43], including one study which compared RBC with a restoration material which does not meet the inclusion criteria for this review [34]. These were categorised within three impact categories: monomer levels in wastewater (*n* = 2) [34, 38], monomer levels in particulate matter (*n* = 1) [43]; and monomer levels in emissions (*n* = 1) [33].

#### Monomer levels in wastewater in studies which include resin-based composite only (*n* = 2)

Two studies reported monomer levels in wastewater for RBC only [38] (specifically, including one without comparison to a relevant restorative material) (see Table S12) [34]. Mourouzis et al. assessed wastewater from clinics which used RBC and polymer infiltrated ceramic restorative material [34]. Testing related specifically to removals, albeit this was simulated by aggravating the restoration material in healthy volunteers, and aimed to detect monomers. Levels of monomers reduced over seven-day time period. Levels of BPA and Triethylene Glycol Dimethacrylate (TEGDMA) were statistically significant both during tests immediately after placement and after seven days. In the only comparative finding between RBC and polymer infiltrated ceramic material, Urethane Dimethacrylate (UDMA) was detected only for the latter material (7.47 ± 0.62 ng/μL).

In the lab setting, Mourouzis et al. assessed the same materials as in the clinic setting using an in vitro test to simulate leaching [34]. TEGDMA was the most commonly detected monomer, and was statistically significant (F(3,36) = 64.119, *p* < 0.001). In the only comparative finding between RBC and polymer infiltrated ceramic CAD/CAM material, as in the clinic setting, UDMA was released only from the polymer infiltrated ceramic material (0.66 ± 0.14 ng/μL).

Polydorou et al. showed that BPA levels in wastewater collected during grinding procedures on RBC was significantly reduced by using filtration materials, but did not report whether this complied with guidance [38].

#### Monomer levels in particulate matter in studies which include resin-based composite only (*n* = 1)

One study considered monomer levels in particulate matter (see Table S13) [43]. Van Landuyt et al. assessed particulate matter in the breathing zone of dentists when working with RBC restorative materials [43]. Tests showed that particles included small methacrylate resin particles which can be linked to monomers, but there was no further analysis of the toxicity for humans [43]. The authors noted that a health relevant metric for the quantification of exposure and dose for nanoscale particles is still under discussion [43]. The lab-based in vitro study component of Van Landuyt et al. provided more detail about the size of particles for different types of RBC material [43]. This study was rated as medium risk of bias [43].

#### Monomer levels in emissions in studies which include resin-based composite only (*n* = 1)

Marquardt et al. assessed methacrylate emission levels during RBC restorations (see Table S14) [33]. Levels of MMA were the highest, followed by HEMA, TEGDMA and EGDMA, but all were reported to be within recommended guidance levels [33]. This study was based in a clinic setting and rated medium level of bias [33].

### Summary of actual versus potential environmental impacts

Of the 21 included studies (across 22 study reports), nine studies reported an actual environmental impact either through (a) assessing compliance with guidance [24, 28, 33, 35, 39, 44, 45] or (b) using a measurable impact, the latter comprising of (i) animal toxicity and (ii) carbon footprint [26, 27, 31]. The remaining 12 studies only showed potential environmental impact due to reporting toxicity levels associated with restorative materials without either assessing compliance with guidance or reporting a measurable impact on the environment [25, 29, 30, 32, 34, 36–38, 40–43]. A summary of these studies with respect to whether or not they measured impact is provided in Table 4.

**Table 4:**
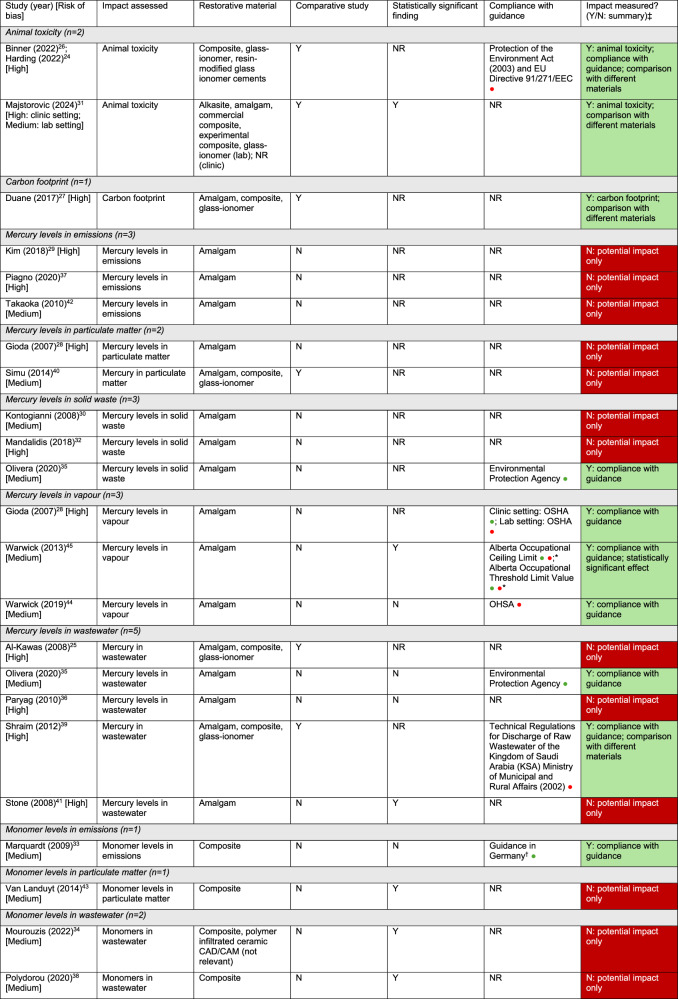
Summary of actual vs potential impact by study and impact category.

*CAD/CAM* computer aided design and computed aided manufacturing, *NR* not reported, *OSHA* Occupational Safety and Health Administration, *USA* United States of America Key: Compliance with guidance: =Yes; =No *Outcome depends on intervention used to reduce vapour emissions exposure. †Specific guidance not reported. ‡ To show a measure of environmental impact (i.e., *actual* rather than *potential* impact), studies were required to either: (a) report compliance with guidance on levels of toxicity in the environment associated with restorative materials, or (b) report effects of toxicity on living organisms or carbon footprint.

## Discussion

We identified 12 impact categories across 21 studies (*n* = 22 study reports) which measured actual or potential environmental impacts relating to dental restorative materials [24–45]. These studies were highly heterogenous in their aims and methods, settings, and in the specific outcomes they reported. They were also limited in terms of the analysis of findings, sometimes only reporting the raw data or descriptive statistical analysis (e.g., averages and standard deviation/error or range of values). Furthermore, none of the studies had a low risk of bias. Due to this heterogeneity and limited analyses, there was limited scope for the synthesis to incorporate a pooled assessment of impact from the available evidence.

Two studies compared the environmental impact of dental amalgam with RBC and GIC restorative materials, but both had limitations which made it difficult to draw definitive conclusions [24, 31]. Majstorovic et al.’s lab-based comparative analysis of animal toxicity showed that dental amalgam was more harmful than either RBC or GIC [31]. However, it was unclear whether this lab-based study used or simulated amalgam separators in limiting the release of amalgam into dental wastewater. Samples from dental clinics in the same study, where amalgam separators were present, contained lower levels of mercury [31]. Contrastingly, Harding et al.’s comparative analysis of dental wastewater from clinic settings which used amalgam separators showed that levels of total suspended solids relating to GIC and RBC were above permissible limits, whereas levels of amalgam were within limits – thus, potentially less toxic [24]. However, in their analysis of animal toxicity, Harding et al. only included RBC and GIC, so we cannot see the relative level of animal toxicity for dental amalgam in this study [24]. To make evidence-based decisions about the environmental impacts of restorative material use, more comparative evidence is required which uses rigorous methods and which clearly shows the relative environmental impacts of different restorative materials in real world settings.

Duane et al.’s comparison of the carbon footprint of dental amalgam, RBC and GIC showed differences in overall carbon footprint and carbon footprint per procedure, but crucially did not include any data relating to the materials themselves, only factors such as the time and energy required to undertake restorations [27].

We are aware of a comparative study by Smith et al., which measures the environmental impact of dental amalgam, RBC and GIC restorative materials from cradle-to-gate, i.e., from processing to packaging [47]. We did not include this study in our analysis because it does not measure impact of restorative activity (i.e., either fillings or removals, or disposal); however, we mention it here because of its broader relevance to the review question. The study found that dental amalgam had the most environmental impact overall, but that RBC material had the most global warming impact [47]. For RBC to be a more environmentally friendly alternative to dental amalgam, Smith et al. recommends findings less intensive energy sources for production [47]. Relatedly, there are many polymer-based materials used in dentistry, similar in composition to RBC materials, where there is a lack of understanding about the extent and nature of environmental impact [48, 49]. It is important that any transition from dental amalgam to RBC materials takes account of this wider evidence-base on the use of polymer-related materials in dentistry, in order to understand the full extent of environmental impact from these materials.

To the best of our knowledge, the most recent systematic review on the environmental impact of amalgam versus non-amalgam restoration materials is the review conducted as part of the CADTH report by Khangura et al. [11]. Only one of the studies overlaps with a study which was identified for the present systematic review, albeit this study was categorised as a health impact study in the sibling review of this paper (in preparation) [50]. As noted above, most of the studies included in the synthesis were literature reviews, commentaries or guidelines which would not meet the inclusion criteria for the present review. The present systematic review has identified studies relevant to environmental outcomes which were not identified by Khangura et al. or not included in the synthesis [11]. Nonetheless, the overall conclusion is broadly the same, i.e., that more evidence is needed to understand the relative environmental impact of amalgam and non-amalgam restoration materials.

### Strengths and limitations

This systematic review presents an updated evaluation of what is known regarding the environmental impacts of a variety of dental restorative materials. The review is based on a systematic search strategy and rigorous screening process, with reference to broad inclusion criteria which were set out in a prospectively registered protocol. We restricted to English-language studies due to lack of translation services, but given the paucity of evidence from across several different North American, European and Asian countries, it is unlikely that this has affected the overall conclusion. The heterogeneity of identified studies limited the scope of the review to synthesise the findings, and there was limited comparative data which was a hinderance to answering the review question regarding the relative impact of dental amalgam versus other materials. The studies were all rated as medium or high risk of bias, which occasionally related to lack of clarity in reporting. Contacting study authors might have resolved some uncertainties about how the studies were conducted. However, we did not have resources to pursue this, and it would have been difficult to follow up potentially missing details in a way which ensured consistent and equal treatment of studies.

### Implications for research and practice

Overall, the current evidence base does not present a clear picture as to the environmental impacts associated with different restorative materials. Further high-quality studies are required. Future research should include meaningful comparisons between the different types of toxicity which are associated with different dental restorative materials, e.g., mercury for dental amalgam, and monomers and other compounds for RBC and GIC. To achieve a meaningful comparison between different restorative materials, it is important to consider either the degree to which levels of toxicity comply with established guidance, or measure toxicity using an approach which can be compared between different materials, e.g., animal toxicity or carbon footprint [26, 31].

Furthermore, we note that mercury in wastewater could be reduced by refraining from removing amalgam fillings to replace them with non-amalgam fillings, as was reported in Shraim et al. [39]. National guidelines such as the American Dental Association do not support removing or replacing amalgam fillings unless required for medical purposes [51]. Additionally, amalgam separators have been shown to reduce mercury levels in wastewater [52], but in some studies in the analysis—including in clinic settings—these were not used [25, 39]. There was also evidence that amalgam separators do not effectively prevent toxic particles from non-amalgam restorative materials entering wastewater [24, 26]. This needs urgent consideration in the context of the trend for transitioning from using amalgam to non-amalgam based restorative materials.

## Conclusions

This report represents the most up to date and comprehensive systematic review of comparative and non-comparative evidence which aimed to understand the potential environmental impacts of amalgam versus non-amalgam restorative materials. Whilst the evidence included in this review indicates that both amalgam and non-amalgam materials are associated with environmental impacts, confidence in these findings is limited due to the heterogeneity of study designs, outcome measures and limited comparative data.

## Supplementary information

Supplementary file

## Data Availability

The data that support the findings of this study are available from the authors upon reasonable request.
