# Supplementary material for: Environmental impact of dental amalgam and alternative restorative materials: a systematic review
Source: BDJ Open. 2026 Jan 21;12:11. doi: 10.1038/s41405-026-00399-z (PMC12824146; doi:10.1038/s41405-026-00399-z)
Supplement: Supplementary file 1 — Supplementary file [file 41405_2026_399_MOESM1_ESM.docx]

**Supplementary file**

*Search report*

Database: MEDLINE
Host: Ovid
Issue: 1946 to November 05, 2024
Date Searched: 6^th^ November 2024
Searcher: SB
Hits: 5604
Strategy:

1. ((canine* or cavity or cavities or caries or dental or dentist* or fill* or incisor* or molar* or premolars or tooth or teeth or repair* or restor*) and amalgam).tw.
2. Dental Amalgam/
3. ("bisphenol A‐Glycidyl methacrylate" or "Bis‐GMA").tw.
4. (resin* and composite* and (canine* or cavity or cavities or caries or dental or dentist* or fill* or incisor* or molar* or premolars or tooth or teeth or repair* or restor*)).tw.
5. ("bulk fill*" and (canine* or cavity or cavities or caries or dental or dentist* or fill* or incisor* or molar* or premolars or tooth or teeth or repair* or restor*)).tw.
6. exp Composite Resins/
7. ("glass ionomer" or "ionomer cement" or giomer*).tw.
8. (GIC and (canine* or cavity or cavities or caries or dental or dentist* or fill* or incisor* or molar* or premolars or tooth or teeth or repair* or restor*)).tw.
9. Glass Ionomer Cements/
10. (compomers or "polyacid modified resin based" or PAMRC).tw.
11. Compomers/
12. or/1-11
13. (ae or co or to or po).fs.
14. (adverse* or bioacc* or bioavail* or breakage* or "clinically accept*" or "clinically unaccpet*" or complication* or discomfort* or harm* or poison* or safe* or "side effect*" or tolerated or tolerance or toxic*).tw.
15. Pregnancy complications/dt
16. ("Bisphenol A" or BPA or mercury).tw.
17. (monomer* adj3 concentration).tw.
18. or/13-17
19. (incidence or prognos* or risk* or survival).tw.
20. exp *Prognosis/
21. exp *risk/
22. exp *Incidence/
23. or/19-22
24. ("global warming" or pollut*).tw.
25. ((biohazard* or dangerous or hazard* or waste) adj4 (chemical* or discharge* or emission* or material* or product* or release or substance*)).tw.
26. (environment* adj4 (affect* or effect* or damag* or hazard* or impact* or sustainab*)).tw.
27. ((carbon or CO2 or CO2e) adj4 (emission* or footprint or impact* or output* or green* or sustainab*)).tw.
28. (greenhouse adj1 (effect* or gas*)).tw.
29. (("life cycle" or lifecycle) adj1 (analysis or analyses or assessment*)).tw.
30. (wastewater or (waste adj2 water)).tw.
31. or/24-30
32. 18 or 23 or 31
33. 12 and 32
34. limit 33 to (english language and yr="2007 -Current")

Database: CAB Abstracts
Host: Ovid
Issue: 1973 to 2024 Week 44
Date Searched: 6^th^ November 2024
Searcher: SB
Hits: 193
Strategy: as per MEDLINE without MeSH terms

Database: CINAHL
Host: EBSCO
Issue: n/a
Date Searched: 6^th^ November 2024
Searcher: SB
Hits: 1425
Strategy:

1. TI ( ((canine* or cavity or cavities or caries or dental or dentist* or fill* or incisor* or molar* or premolars or tooth or teeth or repair* or restor*) and amalgam*) ) OR AB ( ((canine* or cavity or cavities or caries or dental or dentist* or fill* or incisor* or molar* or premolars or tooth or teeth or repair* or restor*) and amalgam*)
2. (MH "Dental Amalgam")
3. TI ( ("bisphenol A‐Glycidyl methacrylate" or "Bis‐GMA") ) OR AB ( ("bisphenol A‐Glycidyl methacrylate" or "Bis‐GMA") )
4. TI ( (resin* and composite* and (canine* or cavity or cavities or caries or dental or dentist* or fill* or incisor* or molar* or premolars or tooth or teeth or repair* or restor*))) OR AB ((resin* and composite* and (canine* or cavity or cavities or caries or dental or dentist* or fill* or incisor* or molar* or premolars or tooth or teeth or repair* or restor*)))
5. (MH "Composite Resins")
6. TI (“bulk fill*” and (canine* or cavity or cavities or caries or dental or dentist* or fill* or incisor* or molar* or premolars or tooth or teeth or repair* or restor*)) OR AB (“bulk fill*” and (canine* or cavity or cavities or caries or dental or dentist* or fill* or incisor* or molar* or premolars or tooth or teeth or repair* or restor*))
7. TI ( ("glass ionomer" or "ionomer cement" or giomer*) ) OR AB ( ("glass ionomer" or "ionomer cement" or giomer*) )
8. (MH "Glass Ionomer Cements")
9. TI ( (GIC and (canine* or cavity or cavities or caries or dental or dentist* or fill* or incisor* or molar* or premolars or tooth or teeth or repair* or restor*)) ) OR AB ( (GIC and (canine* or cavity or cavities or caries or dental or dentist* or fill* or incisor* or molar* or premolars or tooth or teeth or repair* or restor*)) )
10. TI ( (compomers or "polyacid modified resin based" or PAMRC) ) OR AB ( (compomers or "polyacid modified resin based" or PAMRC) )
11. S1 OR S2 OR S3 OR S4 OR S5 OR S6 OR S7 OR S8 OR S9 OR S10
12. TI ( adverse* or bioacc* or bioavail* or breakage* or "clinically accept*" or "clinically unaccpet*" or complication* or discomfort* or harm* or poison* or safe* or "side effect*" or tolerated or tolerance or toxic* ) OR AB ( adverse* or bioacc* or bioavail* or breakage* or "clinically accept*" or "clinically unaccpet*" or complication* or discomfort* or harm* or poison* or safe* or "side effect*" or tolerated or tolerance or toxic* )
13. (MH "Pregnancy Complications/DT")
14. TI ( ("Bisphenol A" or BPA or mercury) ) OR AB ( ("Bisphenol A" or BPA or mercury) )
15. TI (monomer* n2 concentration) OR AB (monomer* n2 concentration)
16. TI ( (incidence or prognos* or risk* or survival) ) OR AB ( (incidence or prognos* or risk* or survival) )
17. (MM "Prognosis+")
18. (MM "Risk Factors")
19. (MM "Incidence")
20. TI ( "global warming" or pollut* ) OR AB ( "global warming" or pollut* )
21. TI ((biohazard* or dangerous or hazard* or waste) n3 (chemical* or discharge* or emission* or material* or product* or release or substance*)) OR AB ((biohazard* or dangerous or hazard* or waste) n3 (chemical* or discharge* or emission* or material* or product* or release or substance*))
22. TI ( environment* n3 (affect* or effect* or damag* or hazard* or impact* or sustainab*) ) OR AB ( environment* n3 (affect* or effect* or damag* or hazard* or impact* or sustainab*) )
23. TI ( (carbon or CO2 or CO2e) n3 (emission* or footprint or impact* or output or green* or sustainab*) ) OR AB ( (carbon or CO2 or CO2e) n3 (emission* or footprint or impact* or output or green* or sustainab*) )
24. TI ( greenhouse n0 (effect or gas*) ) OR AB ( greenhouse n0 (effect or gas*) )
25. TI ( ("life cycle" or lifecycle) n0 (analysis or analyses or assessment*) ) OR AB ( ("life cycle" or lifecycle) n0 (analysis or analyses or assessment*) )
26. TI ( (wastewater or (waste n1 water)) ) OR AB ( (wastewater or (waste n1 water)) )
27. S12 OR S13 OR S14 OR S15 OR S16 OR S17 OR S18 OR S19 OR S20 OR S21 OR S22 OR S23 OR S24 OR S25 OR S26
28. S11 AND S27

Database: Environment Complete
Host: EBSCO
Issue: n/a
Date Searched: 6^th^ November 2024
Searcher: SB
Hits: 518
Strategy:

1. TI ( ((canine* or cavity or cavities or caries or dental or dentist* or fill* or incisor* or molar* or premolars or tooth or teeth or repair* or restor*) and amalgam*) ) OR AB ( ((canine* or cavity or cavities or caries or dental or dentist* or fill* or incisor* or molar* or premolars or tooth or teeth or repair* or restor*) and amalgam*)
2. TI ( ("bisphenol A‐Glycidyl methacrylate" or "Bis‐GMA") ) OR AB ( ("bisphenol A‐Glycidyl methacrylate" or "Bis‐GMA") )
3. TI ((resin* and composite* and (canine* or cavity or cavities or caries or dental or dentist* or fill* or incisor* or molar* or premolars or tooth or teeth or repair* or restor*))) OR AB ( (resin* n3 composite* and (canine* or cavity or cavities or caries or dental or dentist* or fill* or incisor* or molar* or premolars or tooth or teeth or repair* or restor*)))
4. TI (“bulk fill*” and (canine* or cavity or cavities or caries or dental or dentist* or fill* or incisor* or molar* or premolars or tooth or teeth or repair* or restor*)) OR AB (“bulk fill*” and (canine* or cavity or cavities or caries or dental or dentist* or fill* or incisor* or molar* or premolars or tooth or teeth or repair* or restor*))
5. TI ( ("glass ionomer" or "ionomer cement") ) OR AB ( ("glass ionomer" or "ionomer cement") )
6. TI ( (GIC and (canine* or cavity or cavities or caries or dental or dentist* or fill* or incisor* or molar* or premolars or tooth or teeth or repair* or restor*)) ) OR AB ( (GIC and (canine* or cavity or cavities or caries or dental or dentist* or fill* or incisor* or molar* or premolars or tooth or teeth or repair* or restor*)) )
7. TI ( (compomers or "polyacid modified resin based" or PAMRC) ) OR AB ( (compomers or "polyacid modified resin based" or PAMRC) )
8. S1 OR S2 OR S3 OR S4 OR S5 OR S6 OR S7
9. DE "AMALGAMS (Alloys)"
10. DE "SYNTHETIC gums & resins"
11. DE "IONOMERS"
12. S9 OR S10 OR S11
13. TI ( canine* or cavity or cavities or caries or dental or dentist* or fill* or incisor* or molar* or premolars or tooth or teeth or repair* or restor* ) OR AB ( canine* or cavity or cavities or caries or dental or dentist* or fill* or incisor* or molar* or premolars or tooth or teeth or repair* or restor* )
14. S12 AND S13
15. S8 OR S14

Database: GreenFILE
Host: EBSCO
Issue: n/a
Date Searched: 6^th^ November 2024
Searcher: SB
Hits: 213
Strategy:

1. TI ( ((canine* or cavity or cavities or caries or dental or dentist* or fill* or incisor* or molar* or premolars or tooth or teeth or repair* or restor*) and amalgam*) ) OR AB ( ((canine* or cavity or cavities or caries or dental or dentist* or fill* or incisor* or molar* or premolars or tooth or teeth or repair* or restor*) and amalgam*)
2. TI ( ("bisphenol A‐Glycidyl methacrylate" or "Bis‐GMA") ) OR AB ( ("bisphenol A‐Glycidyl methacrylate" or "Bis‐GMA") )
3. TI (resin* and composite*) OR AB (resin* and composite*)
4. TI (“bulk fill*” and (canine* or cavity or cavities or caries or dental or dentist* or fill* or incisor* or molar* or premolars or tooth or teeth or repair* or restor*)) OR AB (“bulk fill*” and (canine* or cavity or cavities or caries or dental or dentist* or fill* or incisor* or molar* or premolars or tooth or teeth or repair* or restor*))
5. TI ( ("glass ionomer" or "ionomer cement") ) OR AB ( ("glass ionomer" or "ionomer cement") )
6. TI ( (GIC and (canine* or cavity or cavities or caries or dental or dentist* or fill* or incisor* or molar* or premolars or tooth or teeth or repair* or restor*)) ) OR AB ( (GIC and (canine* or cavity or cavities or caries or dental or dentist* or fill* or incisor* or molar* or premolars or tooth or teeth or repair* or restor*)) )
7. TI ( (compomers or "polyacid modified resin based" or PAMRC) ) OR AB ( (compomers or "polyacid modified resin based" or PAMRC) )
8. S1 OR S2 OR S3 OR S4 OR S5 OR S6 OR S7

Database: Science Citation Index
Host: Clarivate Analytics
Issue: n/a
Date Searched: 6^th^ November 2024
Searcher: SB
Hits: 5755
Strategy:

1. TS=(((canine* or cavity or cavities or caries or dental or dentist* or fill* or incisor* or molar* or premolars or tooth or teeth or repair* or restor*) and amalgam*))
2. TS=((resin* and composite* and (canine* or cavity or cavities or caries or dental or dentist* or fill* or incisor* or molar* or premolars or tooth or teeth or repair* or restor*)))
3. TS=(((“bulk fill*” and (canine* or cavity or cavities or caries or dental or dentist* or fill* or incisor* or molar* or premolars or tooth or teeth or repair* or restor*)))
4. TS=((GIC and (canine* or cavity or cavities or caries or dental or dentist* or fill* or incisor* or molar* or premolars or tooth or teeth or repair* or restor*
5. TS=(compomers or "polyacid modified resin based" or PAMRC or "bisphenol A‐Glycidyl methacrylate" or "Bis‐GMA" or "glass ionomer" or "ionomer cement" or giomer*)
6. #1 OR #2 OR #3 OR #4 OR #5
7. TI=(adverse* or bioacc* or bioavail* or breakage* or "clinically accept*" or "clinically unaccpet*" or complication* or discomfort* or harm* or poison* or safe* or "side effect*" or tolerated or tolerance or toxic*) OR AB=(adverse* or bioacc* or bioavail* or breakage* or "clinically accept*" or "clinically unaccpet*" or complication* or discomfort* or harm* or poison* or safe* or "side effect*" or tolerated or tolerance or toxic*)
8. TI=(("Bisphenol A" or BPA or mercury)) OR AB=(("Bisphenol A" or BPA or mercury))
9. TI=((monomer* near/2 concentration)) OR AB=((monomer* near/2 concentration))
10. TI=((incidence or prognos* or risk* or survival)) OR AB=((incidence or prognos* or risk* or survival))
11. TI=(("global warming" or pollut*)) OR AB=(("global warming" or pollut*))
12. TI=(((biohazard* or dangerous or hazard* or waste) near/3 (chemical* or discharge* or emission* or material* or product* or release or substance*))) OR AB=(((biohazard* or dangerous or hazard* or waste) near/3 (chemical* or discharge* or emission* or material* or product* or release or substance*)))
13. TI=((environment* near/3 (affect* or effect* or damag* or hazard* or impact* or sustainab*))) OR AB=((environment* near/3 (affect* or effect* or damag* or hazard* or impact* or sustainab*)))
14. TI=(((carbon or CO2 or CO2e) near/2 (emission* or footprint or impact* or output* or green* or sustainab*))) OR AB=(((carbon or CO2 or CO2e) near/2 (emission* or footprint or impact* or output* or green* or sustainab*)))
15. TI=((greenhouse near/0 (effect* or gas*))) OR AB=((greenhouse near/0 (effect* or gas*)))
16. TI=((("life cycle" or lifecycle) near/0 (analysis or analyses or assessment*))) OR AB=((("life cycle" or lifecycle) near/0 (analysis or analyses or assessment*)))
17. TI=(wastewater or (waste near/1 water)) OR AB=(wastewater or (waste near/1 water))
18. #7 OR #8 OR #9 OR #10 OR #11 OR #12 OR #13 OR #14 OR #15 OR #16 OR #17
19. #6 AND #18

Notes: date limited 2007 to date of search.

*Quality appraisal*

Table S1. Quality appraisal scores

| **Study, year, research design** | **Criterion 1: Risk of confounding biases** | **Criterion 2: Risk of post-intervention/exposure selection biases** | **Criterion 3: Risk of misclassified comparison biases (observational studies only)** | **Criterion 4: Risk of performance biases (experimental studies only)** | **Criterion 5: Risk of detection biases** | **Criterion 6: Risk of outcome reporting biases** | **Criterion 7: Risk of outcome assessment biases** | **Overall judgement** |
| --- | --- | --- | --- | --- | --- | --- | --- | --- |
| Al-Kawas, 2008, Cross-sectional with comparator^1^ | High | Medium | Low | NA | Low | Low | Medium | **High** |
| Binner, 2022; Harding 2022, Cross-sectional^2, 3^ | High | Medium | Low | NA | Low | Low | Medium | **High** |
| Duane, 2017, Secondary analysis of dataset^4^ | High | Medium | Low | NA | Medium | Low | Low | **High** |
| Gioda, 2007, Before and after study^5^ | High | Medium | Low | NA | Medium | Low | Low | **High** |
| Kim, 2018, Cross-sectional^6^ | High | Medium | Low | NA | Low | Medium | Medium | **High** |
| Kontogianni, 2008, Cross-sectional survey^7^ | Low | Medium | Low | NA | Medium | Medium | Medium | **Medium** |
| Majstorovic, 2024, Cross-sectional (Part 1: clinic setting)^8^ | High | Medium | Medium | NA | Low | Low | High | **High** |
| Majstorovic, 2024, controlled trial (Part 2: lab-based^8^ | Low | Low | NA | Low | Low | Low | Medium | **Medium** |
| Mandalidis, 2018, Cross-sectional^9^ | High | Low | Low | NA | Low | Low | Low | **High** |
| Marquardt, 2009, Cross-sectional^10^ | Low | Low | Low | NA | Low | Low | Medium | **Medium** |
| Mourouzis, 2022, Controlled trial (clinic and lab setting)^11^ | Medium | Medium | NA | Medium | Low | Medium | Medium | **Medium** |
| Olivera, 2020, Prospective longitudinal^12^ | Low | Low | Low | NA | Low | Low | Medium | **Medium** |
| Paryag, 2010, Modelling study^13^ | Low | Low | Medium | NA | Low | Low | High | **High** |
| Piagno, 2020, Modelling study^14^ | Low | Medium | High | NA | Medium | Medium | High | **High** |
| Polydorou, 2023, Controlled trial^15^ | Low | Low | NA | Low | Low | Low | Medium | Medium |
| Shraim, 2012, Prospective longitudinal with comparator^16^ | High | High | Low | NA | Low | Low | Medium | **High** |
| Simu, 2014, Cross-sectional^17^ | Low | Medium | Medium | NA | Low | Low | Medium | **Medium** |
| Stone, 2008, Prospective longitudinal with control^18^ | High | Medium | Low | NA | Low | Low | Medium | **High** |
| Takaoka, 2010, Modelling study ^19^ | Low | Medium | Low | NA | Low | Low | Medium | **Medium** |
| Van Landuyt, 2014, Controlled trial^20^ | Low | Medium | NA | Medium | Low | Low | Medium | **Medium** |
| Warwick, 2013, Controlled trial (lab based)^21^ | Low | Medium | NA | Low | Low | Low | Medium | **Medium** |
| Warwick, 2019, Prospective longitudinal with control^22^ | Low | Medium | Low | NA | Low | Low | Medium | **Medium** |

Results: supplementary tables S2-S14

Table S2. Toxicity levels in wastewater in studies which compare restorative materials (n=2)

| **Study (Date) [Study design]** | **Restorative material (activity)** | **Exposure (control/comparator)** | **Specific outcomes measured, units** | **Outcome details** |
| --- | --- | --- | --- | --- |
| Clinic setting (n=2) | | | | |
| Al-Kawas (2008) [Cross-sectional with comparator^1^ | Amalgam, composite, glass ionomer (restorations, removals)* | Wastewater from dental chairs at three dental clinics (comparator: different restorative materials used per wastewater sample) | Hg concentration in wastewater samples, mean SD (range), m/L | **Amalgam only samples:** 0.039 mg/L (SD: 0.037; range: 0.004–0.142)  **Amalgam + composite + glass ionomer samples:** 0.024 mg/L (SD: 0.024; range: 0–0.077)  **Composite + glass ionomer samples:** 0.018 mg/L (SD: 0.016; range: 0–0.033)  Guidance compliant: ● |
| Shraim (2011) [Prospective longitudinal with comparator]^16^ | Amalgam, composite, glass ionomer (restorations, removals)^†^ | Wastewater from three dental clinics (1, 2 and 3A, 3B and 3C) (comparator: all clinics use amalgam except only composite at 3C clinic) | Hg mass in samples, average mg per sample, coefficient of variation (CV), range  Comparison of Hg across clinics, mg/L  Hg and other metal concentrations in inlet water, mg | **Hg mass in samples:** Overall average mass per sample: 5.3 mg; CV: 0.0021; range of values of Hg in all samples: 0.009–59.3 mg L^-1^. Most samples contained hazardous levels of metals, including Mg, Mn, Cu, Zn, Sn, Ba, and Hg, many of which are amalgam constituents.  **Clinic comparison:** Clinics 1 and 2, where the most amalgam-based operations are carried out, produced the most Hg (114.2 and 207.1 mg respectively. Clinics 3A and 3B, which treat mostly children and use less amalgam restorations, produced only 9.6 and 12.8 mg Hg respectively. Clinic 3C produced higher Hg content (94.0 mg) than 3A and 3B despite not undertaking amalgam restorations. However, this clinic treats adults who mostly replace Hg amalgam restorations with non-mercury composites and denture operations on amalgam-treated tooth.  **Inlet water Hg and other metals concentration:** Very low metal concentrations, except for Mg (7 mg/L) and Sr (0.1 mg/L). Contribution of inlet water to the metals content of the clinics’ wastewater is negligible, except for Mg, which is relatively high.  Guidance compliant: ● |
| Binner (2022); Harding (2002) [Cross-sectional]^2, 3^ | Amalgam; resin composite, glass ionomer, resin-modified glass ionomer cements (NR) | Wastewater from one dental clinic (NA) | Total suspended solids (TSS), median (range), mg/L  Total dissolved solids (TDS), median (range), g/L | **TSS** Amalgam: 9.83 mg/L (3.33-16.33) Glass-ionomer: 41.67 mg/L (3.30-56.67) Resin composite: 10.00 mg/L (8.89-39.00) Resin modified composite: 2.05 mg/L (NA)  **TDS** Amalgam: 3.36 g/L (1.63-4.41) Glass-ionomer: 20.23 g/L (1.24-39.22) Resin composite: 0.175 g/L (0.15-3.82)  Resin modified composite: 4.07 g/L (NA)  Guidance compliant: ● |

Abbreviations: CV=coefficient of variation; Hg=mercury; NA=not applicable; SD=standard deviation.
Key: Guidance compliant: Yes=● No=● NR=●
* Also root canal, tooth extraction, cavity preparation and temporary restoration, pulpotomy, scaling and polishing, and other.
†Also gingivectomy, crown, bridge, endodontic treatment, pulpectomy, cementation and other

Table S3. Toxicity levels in particulate matter in studies which compare restorative materials (n=1)

| **Study (Year) [Study design]** | **Restorative material (activity)** | **Exposure (control/comparator)** | **Specific outcomes measured, units** | **Outcome details** |
| --- | --- | --- | --- | --- |
| Clinic setting (n=1) | | | | |
| Simu (2014) [Cross-sectional]^17^ | Amalgam, composite, glass ionomer (restorations, removals)* | Exposure to particulate matter in dental clinic setting (comparator: different restorative materials described in analysis) | Size and nature of dental aerosol particles. | Silver amalgam restorations contained the largest number of compounds compared to other types of restoration material, including composite and glass ionomer. Elements of compounds identified during amalgam removals had moderate to high toxicity: Ce, Cs, Gd, Hg, La, Lu, Os, Tl.  Guidance compliant: ● |

Key: Guidance compliant: Yes=● No=● NR=●
*Also, ultrasound scaling, root canal preparation, cavity preparation, teeth preparation for prosthetic purpose, orthodontic controls and other.

Table S4. Animal toxicity in studies which compare restorative materials (n=1)

| **Study (Year) [Study design]** | **Restorative material (activity)** | **Exposure (control/comparator)** | **Specific outcomes measured, units** | **Outcome details** |
| --- | --- | --- | --- | --- |
| Lab setting (n=1) | | | | |
| Majstorovic (2024) [Part 2: Controlled trial]^8^ | Alkasite: Cention Forte  Amalgam  Commercial composite: Tetric EvoCeram  Experimental composite: Bis-GMA/TEGDMA  Glass ionomer cement: Equia Forte HT Fil  (Leaching) | Hg/monomer concentration leached from dental materials in aqueous solution (comparator: amalgam vs non-amalgam materials [i.e. composite vs glass ionomer vs alkasite]; control: E3 medium) | Hg concentration (amalgam samples) after 48h and 7d incubation, µg/L  BPA, TEGDMA and UDMA concentration (non-amalgam samples) after 48h and 7d incubation, mg/mL  Toxicity to zebrafish: 48-hour and 7-day lethal concentrations (LC₅₀ and LC₃₀, g/L) | **Hg concentration (amalgam samples)**: 50.5 µg/L (48h); 4.48 µg/L (7d)  **Monomer concentration (non-amalgam samples):** TEGDMA was detected only in experimental composite and alkasite, both after 48h and 7d. UDMA was detected only in commercial composite after both 48h and 7d. No residual monomers detected from glass ionomer cement. Where detected, concentrations reduced between 48h and 7d.  **Toxicity to zebrafish**  48h: The highest toxicity to zebrafish was observed in experimental composite (i.e. TEGDMA) (LC_50_=0.70 g/L), followed by amalgam (i.e. Hg) (LC_50_=8.27 g/L), commercial composite (i.e. UDMA) (LC_50_=10.94 g/L), glass ionomer (monomer n.d.) (LC_50_=24.84 g/L) and alkasite (i.e. BPA and TEGDMA) (LC_50_=32.22 g/L). Control: ND  7d: LC_50_ values of commercial and experimental composite suspensions were 22.49% and 38.57% higher, respectively, when incubated for 48 h than when incubated for 7d. Samples of glass ionomer and Alkasite incubated for 7d showed no adverse outcomes. Amalgam 314.75 % higher toxicity after 48 h of incubation than after 7d. Control: ND  At 96 hpf both experimental composite and amalgam induced dose-dependent developmental abnormalities, including pericardial edema and scoliosis. This was statistically significant at concentrations corresponding to LC_30_ values for both incubation periods compared control group (p < 0.05). Exposure to commercial composite (both incubation periods) did not result in a statistically significant incidence of developmental alterations (p>0.05), as was the case with glass ionomer and alkasite materials.  Guidance compliant: ● |

Abbreviations: BPA=Bisphenol A; Hg=mercury; hpf=hours post fertilization; LC=lethal concentration; ND=not detected; TEGDMA= Triethylene Glycol Dimethacrylate; UDMA=Urethane Dimethacrylate
Key: Guidance compliant: Yes=● No=● NR=●

Table S5. Carbon footprint in studies which compare restorative materials (n=1)

| **Study (Year) [Study design]** | **Restorative material** | **Total carbon footprint, TCO2e** | **Proportion of dental services, %** | **Carbon footprint per procedure, kgC02e** |
| --- | --- | --- | --- | --- |
| Duane (2017) [Secondary analysis of dataset]^4^ | **Amalgam** | 65,560 | 9.79% | 14.76 |
|  | **Composite** | 63,799 | 9.52% | 14.75 |
|  | **Glass ionomer** | 10,041 | 1.5% | 8.58 |
|  | Guidance compliant: ● | | | |

Abbreviations: kgC02e=kilograms of carbon dioxide equivalent; NA=not applicable; TCO2e=tonnes of carbon dioxide equivalent.
Key: Guidance compliant: Yes=● No=● NR=●

Table S6. Animal toxicity in studies which include multiple materials (with no comparison) (n=2)

| **Study (Year) [Study design]** | **Restorative material (activity)** | **Exposure (control/comparator)** | **Specific outcomes measured, units** | **Outcome details** |
| --- | --- | --- | --- | --- |
| Clinic setting (n=2) | | | | |
| Binner (2022); Harding (2002) [Cross-sectional]^2, 3^ | Resin composite, glass ionomer, resin-modified glass ionomer cements* (NR) | Dental wastewater samples collected from three dental clinics (NA) | 48-hour EC₅₀ for *Daphnia magna*, mL/L | Overall, the EC_50_ ranged from 0.2 to 32.9 mL dental wastewater/L in the three dental practices. Variation in toxicity was attributed to high concentrations of disinfection products in the analysed dental wastewater. These volumes will become diluted as the dental wastewater is released to the sewerage system.  Guidance compliant: ● |
| Majstorovic (2024) [Part 1: Cross-sectional]^8^ | NR^†^ (NR) | Wastewater from dental chair drainage systems (DCDS) for three chairs in one dental clinic (control: E3 medium in lab setting) | Hg/monomer concentration in wastewater samples taken from DCDS with different incubation periods (48h and 7d), µg/L  Toxicity for zebrafish, narrative summary | **Hg concentration:** DCDS 1: 1.86 µg/L (7d); DCDS 2: 0.08 µg/L (48h); DCDS 3: 0.39 µg/L (7d)  **Monomer concentration**: n.d.  **Toxicity for zebrafish:** Exposure of zebrafish to DCDS samples resulted in decreased larval body length and increased occurrences of oedema and blood accumulation. Greatest reduction in body length was for DCDS1 (2.912 µm; p < 0.001), compared to the control group (3.774 µm). Yolk sac oedemas and blood accumulation were recorded on DCDS1 and DCDS3 samples, showing 67.50 % and 45.00 % abnormalities, respectively. Diluting these samples to 50 % reduced toxicity, and no abnormalities were detected at 25 % dilution. No developmental abnormalities were observed in the DCDS2 sample. None of the specimens exposed to DCDS1 hatched at 96 hpf. DCDS2–3 did not impact hatching rates. Control=100% hatching success.  Guidance compliant: ● |

Abbreviations: DCDS=dental chair drainage system; EC=effective concentration; Hg=mercury; NA=not applicable
Key: Guidance compliant: Yes=● No=● NR=●
*Although this study also included amalgam (see Table S2), the test for animal toxicity only included mercury free amalgam materials.
†Dental materials used in clinics not reported, but testing was undertaken for both mercury and monomers. Only mercury was detected in the wastewater, most likely relating to amalgam restorative material, but it is not clear that only amalgam was used in the clinics.

Table S7. Mercury levels in wastewater in studies which include dental amalgam only (n=3)

| **Study (Date) [Study design]** | **Restorative material (activity)** | **Exposure (control/comparator)** | **Specific outcomes measured, units** | **Outcome details** |
| --- | --- | --- | --- | --- |
| Clinic setting (n=1) | | | | |
| Olivera (2020) [Prospective longitudinal]*^12^ | Amalgam (restorations) | Effluent from chairside amalgam separators (CAS) (n=6) at end of service life in military dental centre (NA) | Hg concentrations in effluent of CAS, mg/L (mean and range) | **Mean CAS effluent Hg concentration:** 0.95 +/- 0.89 mg/L to 4.15 +/- 2.51 mg/L.  **Minimum and maximum Hg concentrations measured in CAS effluent grab samples:** 0.05 mg/L and 11.93 mg/L. respectively, and mean concentration was 1.99 +/- 2.09 mg/L  Guidance compliant: ● |
| Lab setting (n=1) | | | | |
| Paryag (2010) [Modelling study]^13^ | Amalgam (removals) | Concentration of Hg in filtrate from wastewater and solid sample in lab setting (NA) | Hg discharge from amalgam restoration removal in Trinidad and Tobago, mean g/month and g/day | **All Dentists in Trinidad and Tobago for 1 month:** 22285.35 g/month  **Per Dentist per month:** 94.82 g/month  **Per Dentist per day:** 3.4 g/day  Guidance compliant: ● |
| Clinic/lab setting combined (n=1) | | | | |
| Stone (2009) [Prospective longitudinal with control]^18^ | Amalgam (NR) | Clinic: Chairside wastewater from dental chairs fitted with air/water separator (n=5)  Lab: lab-prepared samples  (Control: de-ionized water) | Hg level from wastewater exposed to chlorine vs monochloramine (1 mg/L and 10 mg/L)  T-test of mean natural log-transformed mercury concentration, ln(Hg), µg/L  ANCOVA | **Hg concentration:** 10 mg/L chlorine: 0.59 Hg mg/L (mean) (n= 25, SD= 1.06). 1 mg/L chloramine: 0.023 Hg mg/L (mean) (n= 25, SD = 0.010). 10 mg/L chloramine: 0.024 Hg mg/L (mean) (n= 25, SD = 0.011). Control: 0.013 Hg mg/L (mean) (n=19, SD=0.006  **T-tests:** Statistically significant difference in the natural log-transformed mercury (Hg) concentrations between 10 mg/L chlorine and both 1 mg/L and 10 mg/L monochloramine treatments. E.g. the mean ln(Hg) for 10 mg/L chlorine was −1.52 (SD: 1.34), compared to −3.87 (SD: 0.56) for 10 mg/L monochloramine. This difference was statistically significant (p < 0.001), indicating that significantly higher levels of mercury were observed following treatment with 10 mg/L chlorine compared to 10 mg/L monochloramine.  **ANCOVA results**: Each one of the independent variables was significantly related to the Hg concentration: treatment, sample (laboratory-generated vs. clinical amalgam), time and pH all significant (p<0.001). Results demonstrate that chloramine does not mobilize Hg to the same degree as chlorine.  Guidance compliant: ● |

Abbreviations: ANCOVA=analysis of covariance; CAS=chairside amalgam separator; Hg=mercury; NA=not applicable; NR=not reported
Key: Guidance compliant: Yes=● No=● NR=●
*Unlike other studies assessing guidance compliance, which focus on assessments in relation to Hg levels, Olivera et al. assess guidance compliance in relation to CAS performance.

Table S8. Mercury levels in solid filtrate/solid waste in studies which include dental amalgam only (n=3)

| **Study (Year) [Study design]** | **Restorative material (activity)** | **Exposure (control/comparator)** | **Specific outcomes measured, units** | **Outcome details** |
| --- | --- | --- | --- | --- |
| Clinic setting (n=3) | | | | |
| Kontogianni (2007) [Cross-sectional survey]^7^ | Amalgam (NR) | Disposal of solid amalgam waste in Thessaloniki, Greece (comparison: % total amalgam waste vs [other] metal-bearing and non-metal bearing waste) | Total amalgam waste per year (vs metal-bearing vs non-metal bearing), % and g  Mean daily disposal of solid amalgam waste per clinic, g | **% of total waste per year:** 0.1% (amalgam) vs 15.12% (metal bearing) vs 84.78% (non-metal bearing)  **Daily disposal per clinic:** 5g  Guidance compliant: ● |
| Mandalidis (2018) [Cross-sectional]^* 9^ | Amalgam (removals, restorations) | Disposal of solid amalgam waste in Xanthi, Greece (NA) | Overall waste production, %  Daily waste production per practice, g | **Overall waste production:**  Excess amalgam: 0.01%  Plastic amalgam capsules: 0.03%  Daily waste production per practice:  Excess amalgam: 0.02g ± 0.01  Plastic amalgam capsules: 0.1g ± 0.04  Guidance compliant: ● |
| Olivera (2020) [Prospective longitudinal] ^†12^ | Amalgam (restorations) | Solid filtrate from chairside amalgam separators (CAS) (n=6) at end of service life in military dental centre (NA) | Material in filtrate, % and g  Hg in solid filtrate of CAS, % | **Material in filtrate:** 57% non-amalgam materials (145.422 g); 43% amalgam (109.511 g).   **Hg in filtrate:** Hg (20.9%) was the major metal of the recovered solids, with silver (11.7%), tin (6.1%), and copper (4.3%) making up the remaining amalgam constituent.  Guidance compliant: ● |

Abbreviations: CAS=Chairside amalgam separator; Hg=mercury; NA=not applicable; NR=not reported; %w/w=weight/weight percentage.
Key: Guidance compliant: Yes=● No=● NR=●
*Includes solid waste without measuring mercury concentration; included here because it is implied that this waste is related to environmental impact from mercury levels in waste.
†Unlike other studies assessing guidance compliance, which focus on assessments in relation to Hg levels, Olivera et al. assess guidance compliance in relation to CAS performance.

Table S9. Mercury levels in emissions in studies which include dental amalgam only (n=3)

| Study (Year) [Study design] | Restorative material (activity) | Exposure (control/comparator) | Specific outcomes measured, units | Outcome details |
| --- | --- | --- | --- | --- |
| Clinic setting (n=1) | | | | |
| Kim (2018) [Cross-sectional with modelling]^6^ | Amalgam (incineration of extracted teeth) | Emissions from general dental clinics (n=2) and private dental clinics (n=5) (NA) | Hg levels in emissions per year (total and adjusted estimate for dental institution weights in Korean dentistry units), kg | Total emissions: 48.86 kg (95% confidence interval [CI] 41.53–58.63 kg) of Hg incinerated with extracted teeth.  Adjusted estimate for dental institution weights: 42.53 kg (95% CI 34.11–52.17 kg).  Guidance compliant: ● |
| Crematorium setting (n=2) | | | | |
| Piagno (2020) [Modelling study]^14^ | Amalgam (incineration of extracted teeth) | Emissions from crematoriums in BC, Canada (NA) | Estimated annual Hg emissions from cremations, kg/year  Overall Hg contribution to emissions in BC, % | Estimated annual emissions: 35.8 kg/year  Overall %: >7% of overall emissions of Hg to the atmosphere in BC.  Guidance compliant: ● |
| Takaoka (2010) [Modelling study]^19^ | Amalgam (incineration of extracted teeth) | Emissions from crematoriums in Japan (NA) | Estimated annual Hg emissions from cremations, kg/year  Overall Hg contribution to emissions in Japan, % | Estimated annual emissions: 35.1 kg/year  Overall %: <0.01% of the total amount of Hg released into the atmosphere in Japan.  Guidance compliant: ● |

Abbreviations: BC=British Columbia; NA=not applicable; NR=not reported; OSHA= Occupational Safety and Health Administration SE=standard error
Key: Guidance compliant: Yes=● No=● NR=●

Table S10. Mercury levels in vapour in studies which include dental amalgam only (n=3)

| Study (Year) [Study design] | Restorative material (activity) | Exposure (control/comparator) | Specific outcomes measured, units | Outcome details |
| --- | --- | --- | --- | --- |
| Clinic setting (n=2) | | | | |
| Gioda (2007) [Before and after study]*^5^ | Amalgam (restorations, removals) | Vapour from students undertaking dental exam in clinic setting (comparator: student vs professors observing exam, stationary measurements inside vs outside building) | Hg levels in vapour per student and professors, μg/m^3^ (mean and SE)  Hg levels in vapour in room and outside building (baseline reference), μg/m^3^ (mean and SE) | **People in room:**  Students: 50.6 (mean) μg/m^3^ (+/- 9.7 SE);  Professors: 39.7 (mean) μg/m^3^ (+/-6.4 SE).  **Stationary samplers in/outside room:**  Indoor stationary sampler: 24.2 μg/m^3^ (+/- 3.1 SE);  Outdoor indoor stationary sampler: 10.9 μg/m^3^  Guidance compliant: ●^†^ |
| Warwick (2019) [Prospective longitudinal with control]^22^ | Amalgam (removals) | Concentration of Hg vapour after exposure to particulate matter during restoration removal (control: no amalgam removal) | Peak Hg Vapour, μg/m^3^  15 min average Hg vapour, μg/m^3^  30 min average Hg vapour, μg/m^3^  60 min average Hg vapour, μg/m^3^ | Peak Hg vapour: 195 μg/m^3^ (mean) (241 SD), Median: 80μg/m3  15 min average Hg vapour:152 μg/m^3^ (mean) (217 SD), Median: 46μg/m3  30 min average Hg vapour: Mean 135 μg/m^3^ (196 SD), Median: 43μg/m3  60 min average Hg vapour: Mean: 109μg/m3 (163 SD), Median: 26 μg/m3  Control: <detectable μg of Hg mass  Guidance compliant: ● |
| Lab setting (n=2) | | | | |
| Gioda (2007) [Before and after study]*^5^ | Amalgam (restorations, removals) | Vapour from students undertaking dental exam in lab setting (NA) | Hg levels per student, mg/m^3^ (median and range) | 2.2 mg/m^3^ (median) (range: 1.1-3.3)  Guidance compliant: ● |
| Warwick (2013) [Controlled trial]^21^ | Amalgam (removals) | Vapour from removals with simultaneous use of water spray and suction vs with suction only (control: with neither suction nor water spray) | Hg levels in vapour, mean and SD (μg/m^3^); median and range (μg/m^3^); statistical significance | **With water spray and suction:** 8.0 μg/m^3^ (mean) ± 3.7 (SD); 8.0 μg/m^3^ (median) (range: 4.0–19.0)  Guidance compliant: ●  **With suction only:** 142.0 μg/m^3^ (mean) ± 234.6 (SD); 68.0 μg/m^3^ (median) (range: 14.0–999.0); exposure greater than suction & water statistically significant (p < 0.001)  Guidance compliant: ●  **With no water spray or suction:** 214 μg/m^3^ (mean) ± 226.4 (SD); 117.0 μg/m^3^ (median) (range: 34.0–796.0); exposure greater than suction and water statistically significant (p = 0.031)  Guidance compliant: ● |

Abbreviations: ACGIH=American Conference of Governmental Industrial Hygienists; Hg=mercury; OSHA=Occupational Safety and Health Administration; NA=not applicable; SD=standard deviation; SE=standard error
Key: Guidance compliant: Yes=● No=● NR=●
*Does not include pre-post mercury level comparison for vapour levels (this is limited to the particulate matter component of the study, see table below).
†Compliant with US Occupational Safety and Health Administration (OSHA) permissible exposure limit (100 μg/m3) but slightly above the advisory American Conference of Governmental Industrial Hygienists (ACGIH) time-weighted average (TWA) 8h day/40h week recommendation (i.e. 50 μg/m3), although students are only exposed for 4.2 hours on one day.

Table S11. Mercury levels in particulate matter (PM) in studies which include dental amalgam only (n=1)

| **Study (Year) [Study design]** | **Restorative material (activity)** | **Exposure (control/comparator)** | **Specific outcomes measured, units** | **Outcome details** |
| --- | --- | --- | --- | --- |
| Clinic setting (n=1) | | | | |
| Gioda (2007) [Before and after study]^5^ | Amalgam (restorations, removals) | PM_10_ from students undertaking dental exam in clinic setting (comparator: particulate matter before exam) | Overall levels of PM_10_; Hg bound to PM_10_, μg/m^3^ | Two days before exam: 35.6 (ug/m^3^); Hg: 0.02 (ug/m^3^)  Exam day: 68.2 (ug/m^3^); Hg: 0.2 (ug/m^3^)  Three days after exam: 35.0 (ug/m^3^); Hg: 0.01 (ug/m^3^)  Guidance compliant: ● |
| Lab setting (n=1) | | | | |
| Gioda (2007) [Before and after study]^5^ | Amalgam (restorations, removals) | PM_10_ from students undertaking dental exam in lab setting (comparator: particulate matter before exam) | Overall levels of PM_10_; Hg bound to PM_10_, μg/m^3^ | Day 1: (no activity): 9.2 μg/m^3^; ND  Day 2: (preparation): 39.8 μg/m^3^ ND  Day 3: (restorations): 12.2 μg/m^3^; 0.2 μg/m^3^  Day 4: (removing and restorations): 16.2 μg/m^3^; 0.1 μg/m^3^  Day 5: (removing and restorations): 12.4 ug/m^3^:1.1 μg/m^3^  Day 6: (removing and restorations, cleaning lab):41.6 μg/m^3^; 0.9 μg/m^3^  Guidance compliant: ● |

Abbreviations: ND=not detected; PM=particulate matter

Key: Guidance compliant: Yes=● No=● NR=●

Table S12. Monomer levels in wastewater in studies which included resin-based composite only (n=2)

| Study (Year) [Study design] | Restorative material (activity) | Exposure (control/comparator) | Specific outcomes measured, units | Outcome details |
| --- | --- | --- | --- | --- |
| Clinic setting (n=1) | | | | |
| Mourouzis (2022) [Controlled trial]^11^ | Composites: Clearfil Majesty Posterior PLT; Clearfil Photo Core PLT  Polymer infiltrated ceramic CAD/CAM: Vita Enamic® [not relevant]  (removals)* | Wastewater from dental unit (control: clean dental unit wastewater; comparator: different composite materials and polymer infiltrated ceramic [not relevant]) | BPA, Bis-GMA, TEGDMA and UDMA at two time points: immediately after placement and 7d, ng/μL | Immediately after placement: BPA and TEGDMA monomers showed statistically significant results for the materials tested (F(3,36) = 37.990 and F(3,36) = 54.441, p < 0.001, respectively). The highest levels of TEGDMA were released by Clearfil Majesty Posterior PLT material (1.35 ± 0.52 ng/μL) and this was statistically significant compared to other materials (p < 0.001). UDMA was detected only for Vita Enamic® material (7.47 ± 0.62 ng/μL). Control: ND  7d timepoint: BPA and TEGDMA monomers showed statistically significant results (F(3,36) = 6.760 and F(3,36) = 4.176, p< 0.001, respectively). Pairwise comparisons between different group materials for all the released monomers showed that Clearfil Majesty Posterior PLT material released the highest levels of TEGDMA (1.31 ±0.58 ng/μL) and this was statistically significant compared to other materials (p < 0.001). UDMA and Bis-GMA were not detected in any material after 7d. Control: ND  Guidance compliant: ● |
| Lab setting (n=2) | | | | |
| Mourouzis (2022) [Controlled trial]^11^ | Composites: Clearfil Majesty Posterior PLT; Clearfil Photo Core PLT  Polymer infiltrated ceramic CAD/CAM: Vita Enamic® [not relevant]  (removals)^†^ | Wastewater from dental unit collected during in vitro experiment (control: clean dental unit wastewater) | BPA, Bis-GMA, TEGDMA and UDMA, ng/μL | BPA was detected in the wastewater samples only for the high-viscosity core build-up composite material (0.11 ± 0.02 ng/μL). TEGDMA was detected in the wastewater samples of all the materials. TEGDMA monomer yielded statistically significant results (F(3,36) = 64.119, p < 0.001). Pairwise comparisons between Clearfil Majesty Posterior PLT and Clearfil PhotoCore PLT revealed that Clearfil Majesty Posterior PLT material released the highest amount of TEGDMA (1.07 ± 0.19 ng/μL) and the result was statistically significant (p < 0.001). Between the CAD/CAM material Vita Enamic® and the Clearfil Photo Core PLT material, no statistically significant result was identified (p > 0.05) for the amount of TEGDMA. UDMA was released only from the Vita Enamic® CAD/CAM material (0.66 ± 0.14 ng/μL). Control: ND  Guidance compliant: ● |
| Polydorou (2020) [Controlled trial]^15^ | Ormocer: Ceram X®  Nanofilled resin composite: FiltekTM Supreme XTE  Dual-cure core build-up material: Core-X® flow  (Dental grinding procedures) | Wastewater from dental unit collected during in vitro experiment (control: clean dental unit wastewater) | BPA before filtration, mg/L  BPA after filtration (filtration materials: Zeosorb; Katalox Light; Catalytic Carbon), mg/L | Significant difference in BPA reduction observed for three filtration materials (p < 0.0001). Paired t-test analysis showed significant reduction in BPA amount after filtration for all the materials (p < 0.05). However, filtration with Zeosorb and Katalox Light resulted in only small reduction of BPA, while treatment with Catalytic Carbon resulted in a clinically relevant reduction of BPA by 99.3% (p < 0.05).  Guidance compliant: ● |

Abbreviations: Bis-GMA=Bisphenol A-glycidyl methacrylate; BPA=Bisphenol A; CAD/CAM=Computer-Aided Design/Computer-Aided Manufacturing; ND=not detected; TEGDMA= Triethylene Glycol Dimethacrylate; UDMA=Urethane Dimethacrylate

Key: Guidance compliant: Yes=● No=● NR=●

*Samples in healthy volunteers were milled to simulate the clinical operations of polishing resin composite restorations or the grinding and removal of failed composite resin restorations.

†Also, simulation of polishing resin composite restorations.

Table S13. Monomer levels in particulate matter in studies which include composite only (n=1)

| Study (Year) [Study design] | Restorative material (activity) | Exposure (control/comparator) | Specific outcomes measured, units | Outcome details |
| --- | --- | --- | --- | --- |
| Clinic setting (n=1) | | | | |
| Van Landuyt (2014) [Controlled trial]^20^ | Composite (restorations)* | Abrasive procedures relating to composites in clinic setting (NA) | Concentrations of nanoparticles, particle size | Peak moments of high concentrations of nanoparticles in breathing zone of the dentist and patient were associated with abrasive procedures of composites. Reshaping and contouring of composites resulted in peak concentrations between 0.1 and 1 x 106 cm-3 in the breathing zone of the dentist. Background measurements remained stable during these peaks between 5000 and 10,000 cm^3^.  Guidance compliant: ● |
| Lab setting (n=1) | | | | |
| Van Landuyt (2014) [Controlled trial]^20^ | Composite (grinding material to simulate abrasive procedures, including restorations) | Abrasive procedures relating to composites in clinic setting (NA) | Concentrations of nanoparticles, nm | Kruskal–Wallis analysis showed Gradia Direct and Tetric EvoCeram (Ivoclar-Vivadent, Schaan, Liechtenstein) produced statistically larger particles (p < 0.05) than Filtek Supreme XTE (3M ESPE), GrandiO and Z100 (3M ESPE).  Nanoparticles released by the latter three composites were not statistically different in size. When measured after 1, 4 or 7 min the mean particle diameter was statistically different for all composites indicating that all composites had an individual agglomeration tendency.  Guidance compliant: ● |

Abbreviations: NA=not applicable; nm=na
Key: Guidance compliant: Yes=● No=● NR=●
*Also, reshaping and contouring of composites.

Table S14. Monomer levels in emissions in studies which include resin-based composite only (n=1)

| Study (Year) [Study design] | Restorative material (activity) | Exposure (control/comparator) | Specific outcomes measured, units | Outcome details |
| --- | --- | --- | --- | --- |
| Clinic setting (n=1) | | | | |
| Marquardt (2009) [Cross-sectional]^10^ | Composite (restorations) | Air sampling during the treatment of patients at four dental practices (NA) | Measurements of methacrylates during composite restoration treatments | Methyl methacrylate (MMA) concentrations (=0.4 mg/m^3^) were 10 times higher than HEMA and TEGDMA, and 30 times higher than EGDMA. Levels of MMA were lower than the maximum allowable concentration value and short-term threshold values (reported as 210 mg/m^3^ in Germany between 100 and 400 mg/m^3^ in other countries). Toxicological risk for dental personnel reported as minor. No maximum allowable concentration values are available for HEMA, EGDMA, or TEGDMA.  Guidance compliant: ● |

Abbreviations: EGDMA=Ethylene Glycol Dimethacrylate; HEMA=2-Hydroxyethyl Methacrylate; MMA=methyl methacrylate; NA=not applicable; TEGDMA=Triethylene Glycol Dimethacrylate
Key: Guidance compliant: Yes=● No=● NR=●

*Supplementary file references*

1. Al-Kawas S, Abu-Yousef IA, Kanan S, et al. Analysis of mercury in wastewater of some dental clinics in United Arab Emirates. Journal of International Environmental Application & Science. 2008;3(1):21-8.

2. Binner H, Kamali N, Harding M, Sullivan T. Characteristics of wastewater originating from dental practices using predominantly mercury-free dental materials. Sci Total Environ. 2022;814:152632.

3. Harding M, Sullivan T, Binner H, et al. Assessment of the Environmental and Health Impacts Arising from Mercury-free Dental Restorative Materials. University College Cork; 2022.

4. Duane B, Lee MB, White S, et al. An estimated carbon footprint of NHS primary dental care within England. How can dentistry be more environmentally sustainable? Br Dent J. 2017;223(8):589-93.

5. Gioda A, Hanke G, Elias-Boneta A, Jimenez-Velez B. A pilot study to determine mercury exposure through vapor and bound to PM10 in a dental school environment. Toxicol Ind Health. 2007;23(2):103-13.

6. Kim HJ, Park JH, Sakong J. Estimation of Mercury Emission from Incineration of Extracted Teeth with Dental Amalgam Fillings in South Korea. Int J Environ Res Public Health. 2018;15(7).

7. Kontogianni S, Xirogiannopoulou A, Karagiannidis A. Investigating solid waste production and associated management practices in private dental units. Waste Manag. 2008;28(8):1441-8.

8. Majstorovic M, Babic Brcic S, Malev O, et al. Environmental implications of dental restorative materials on the zebrafish Danio rerio: Are dental chair drainage systems an emerging environmental threat? Environ Toxicol Pharmacol. 2024;110:104499.

9. Mandalidis A, Topalidis A, Voudrias EA, Iosifidis N. Composition, production rate and characterization of Greek dental solid waste. Waste Manag. 2018;75:124-30.

10. Marquardt W, Seiss M, Hickel R, Reichl FX. Volatile methacrylates in dental practices. J Adhes Dent. 2009;11(2):101-7.

11. Mourouzis P, Andreasidou E, Arhakis A, et al. Release of monomers in dental wastewater during treatment. A comparative in vitro and in vivo study based on Fabric phase Sorptive extraction. Microchemical Journal. 2022;183.

12. Olivera DS, Morgan MT, Tewolde SN, et al. Clinical Evaluation of a Chairside Amalgam Separator to Meet Environmental Protection Agency Dental Wastewater Regulatory Compliance. Oper Dent. 2020;45(2):151-62.

13. Paryag A, Paryag AS, Rafeek RN, Pilgrim A. Mercury pollution from dental amalgam waste in Trinidad and Tobago. Journal of Water Resource and Protection. 2010;2(8):762-9.

14. Piagno H, Afshari R. Mercury from crematoriums: human health risk assessment and estimate of total emissions in British Columbia. Can J Public Health. 2020;111(6):1011-9.

15. Polydorou O, Schmidt OC, Spraul M, et al. Detection of Bisphenol A in dental wastewater after grinding of dental resin composites. Dent Mater. 2020;36(8):1009-18.

16. Shraim A, Alsuhaimi A, Al-Thakafy JT. Dental clinics: a point pollution source, not only of mercury but also of other amalgam constituents. Chemosphere. 2011;84(8):1133-9.

17. Simu MR, Borzan C, Mesaros M, et al. COMPLEX CHARACTERIZATION OF DENTAL OFFICE AEROSOLS REVEALS IMPORTANT LOADS OF RISK ELEMENTS FOR THE HUMAN HEALTH. DIGEST JOURNAL OF NANOMATERIALS AND BIOSTRUCTURES. 2014;9(4):1429-38.

18. Stone ME, Scott JW, Schultz ST, et al. Comparison of chlorine and chloramine in the release of mercury from dental amalgam. Sci Total Environ. 2009;407(2):770-5.

19. Takaoka M, Oshita K, Takeda N, Morisawa S. Mercury emission from crematories in Japan. Atmospheric Chemistry & Physics Discussions. 2009;9(6):27195-214.

20. Van Landuyt KL, Hellack B, Van Meerbeek B, et al. Nanoparticle release from dental composites. Acta Biomater. 2014;10(1):365-74.

21. Warwick R, O'Connor A, Lamey B. Mercury vapour exposure during dental student training in amalgam removal. J Occup Med Toxicol. 2013;8(1):27.

22. Warwick D, Young M, Palmer J, Ermel RW. Mercury vapor volatilization from particulate generated from dental amalgam removal with a high-speed dental drill - a significant source of exposure. J Occup Med Toxicol. 2019;14(1):22.
